# Araneae Sloveniae: a national spider species checklist

**DOI:** 10.3897/zookeys.474.8474

**Published:** 2015-01-21

**Authors:** Rok Kostanjšek, Matjaž Kuntner

**Affiliations:** 1 Department of Biology, University of Ljubljana, Slovenia; 2 Institute of Biology, Scientific Research Centre, Slovenian Academy of Sciences and Arts, Ljubljana, Slovenia; 3 Centre for Behavioural Ecology & Evolution, College of Life Sciences, Hubei University, Wuhan, China; 4 National Museum of Natural History, Smithsonian Institution, Washington, DC, USA

**Keywords:** Faunistics, ecology, regional faunas, Slovenia, Palearctic, species richness

## Abstract

The research of the spider fauna of Slovenia dates back to the very beginning of binomial nomenclature, and has gone through more and less prolific phases with authors concentrating on taxonomy, faunistics, ecology and zoogeographic reviews. Although the body of published works is remarkable for a small nation, the faunistic data has remained too scattered for a thorough understanding of regional biotic diversity, for comparative and ecological research, and for informed conservation purposes. A national checklist is long overdue. Here, a critical review of all published records in any language is provided. The species list currently comprises 738 species, is published online at http://www.bioportal.si/katalog/araneae.php under the title Araneae Sloveniae, and will be updated in due course. This tool will fill the void in cataloguing regional spider faunas and will facilitate further araneological research in central and southern Europe.

## Introduction

At roughly 20,000 km^2^, Slovenia’s territory may be relatively small, but its position in between the Mediterranean, Alpine, Dinaric and Pannonic biogeographic regions is unique, and consequently, its native biota is rich and remarkable. Despite several active episodes of spider research in Slovenia, reviewed by Kuntner and Šereg (2002), a comprehensive national spider checklist has not been available, yet is long overdue. Our aim is to provide a critically filtered list of all relevant literature, in any language, that listed data relevant for Slovenian spider faunistics, and a list of all spider species recorded in this territory, cleaned for dubious, erroneous, and synonymous records. This checklist is simultaneously published online facilitating regular updates.

## Historic setting

The beginnings go back to the 18^th^ century, when Scopoli (1763) described 44 spider species from Carniola following Linné’s nomenclature. Scopoli’s cataloguing (Scopoli 1763, 1772) included both plants and animals, with spiders taking a prominent part. Most of the 19^th^ century works were also taxonomic and cataloguing in nature (Damin 1900; Doblika 1853; Doleschall 1852; Hamann 1896; Joseph 1881, 1882; Schiödte 1847; Simon 1868, 1882), adding to the recognition of the territory’s biotic importance, particularly in karstic caves (Hamann 1896; Schiödte 1847).

Early 20^th^ century brought more taxonomic investigations and numerous species descriptions. Again, cave faunas played a prominent role and Slovenia’s karst (a word deriving from the geographic name Kras) became the cradle of subterranean biology in large part due to lively araneological research (Absolon and Kratochvíl 1932, 1933; Bohinec 1926; Caporiacco 1938; Fage 1919, 1931; Kratochvíl 1933, 1934, 1936, 1939, 1948; Kratochvíl and Miller 1940; Megušar 1914; Roewer 1931; Wichmann 1926). These and other authors from the first half of 20^th^ century also documented the epigeic spider fauna (Caporiacco 1949; Drenski 1936; Giltay 1932; Hafner 1925; Kratochvíl 1935; Kulczynski 1915; Miller 1940; Reimoser 1919; Šilhavy 1944).

The second part of the 20^th^ century saw numerous important taxonomic additions, either stand alone or within revisions. Among them, several authors stand out as highly influential. Paolo Marcello Brignoli, as an example, revised numerous genera from several families contributing to the Mediterranean spider taxonomy (Brignoli 1971a, b, 1975, 1976, 1978, 1980). In her numerous revisions, Christa Deeleman-Reinhold provided a solid basis for understanding cave and surface linyphiids and dysderids from Slovenia and the Balkans (Deeleman-Reinhold 1971a, b, 1973a, b, 1975, 1977a, b, 1978, 1980, 1981, 1983, 1985, 1993; Deeleman-Reinhold and Deeleman 1988). Furthermore, Hermann Wiehle contributed with descriptions of new Slovenian taxa (Wiehle 1961, 1964), and Konrad Thaler published numerous regional, particularly Alpine, revisions encompassing several families (Thaler 1972, 1973, 1976, 1978a, b, 1981a, b, 1982, 1986, 1990a, b, c, 1994, 1997; Thaler and Buchar 1994; Thaler and Polenec 1974). In addition, other contemporary authors also added to taxonomic understanding of the regional fauna (Maurer 1982a, b; Miller and Polenec 1975a, b; Millidge 1979; Pesarini 1989; Tongiorgi 1966; Van Helsdingen 1969; Vanuytven et al. 1994; Wunderlich 1980).

However, this time should also be known as the “Polenec era” (Kuntner and Šereg 2002). Although Anton Polenec only touched on taxonomy, his several decades-long investigations of Slovenia’s spiders have made a lasting imprint on the faunistics and community ecology, in particular of ground spider assemblages. Polenec published numerous sampling checklists and/or observations (Polenec 1952, 1954, 1957, 1958, 1959, 1960, 1961a, b, c, d, 1962a, b, c, 1963a, b, c, d, e, 1964a, b, 1965a, b, 1966a, b, 1967a, b, 1968a, b, 1969a, b, c, 1970a, b, 1971a, b, c, 1972, 1973a, b, c, d, e, 1974a, b, 1975a, b, 1976a, b, 1977, 1978a, b, c, d, 1979a, b, 1980a, b, 1981, 1982a, b, 1983, 1984, 1985, 1986, 1987, 1988, 1989, 1992; Polenec and Thaler 1980). He also coauthored the only catalogue of Yugoslav spider fauna (Nikolić and Polenec 1981), where the republic of Slovenia was within the same geographic borders as today.

Other authors of the end of the 20^th^ century have also contributed to the overall understanding of spider species distributions in Slovenia (Alicata 1966; Balarin 1984; Bedjanič 1999; Blick et al. 1995; Bole 1974; Bole et al. 1980, 1982, 1993; Bosmans 1997; Brenčič 1999; Buchar and Polenec 1974; Deltshev 1999; Drovenik 1971; Fuhn and Polenec 1967; Gasparo 1995, 1996, 2000; Gasparo and Thaler 2000; Grimm 1985, 1986; Kiauta 1960; Knoflach 1994, 1996; Knoflach and Thaler 2000; Komposch and Steinberger 1999; Kranjc and Novak 1977; Kratochvíl 1970; Kuntner 1994, 1996, 1997a, b, c, d, 1998, 1999a, b; Kuntner and Baxter 1997; Kuntner et al. 1999a, 1999b; Mršić 1997; Nikolić 1957, 1961, 1963; Novak 1967, 1971, 1981, 2005a; Novak et al. 1981; Novak 1982; Novak and Sivec 1976; Pesarini 1989; Polak 1994, 1997; Růžička et al. 2005; Sket 1979, 1993, 1999, 2000; Spazzapan-Brelih 1964; Töpfer-Hofmann et al. 2000; Zingerle 1999; Zupančič 1984).

The 21^st^ century began with some higher level systematics and taxonomic works that used Slovenia’s exemplars (Agnarson 2004; Arnedo et al. 2004, 2009; Gregorič 2008; Kuntner et al. 2008; Řezáč et al. 2008a, b; Van Helsdingen et al. 2001; Wang et al. 2010), but mostly continued to document the fauna through faunistic contributions (Buchar and Dolansky 2011; Buchar and Thaler 2002; Čandek et al. 2013, 2015; Decae 2010; Fišer and Kostanjšek 2001; Franc 2004; Gorjan and Fišer 2010; Gregorič and Kuntner 2009; Kostanjšek 2000, 2001, 2002a, b, 2003, 2004a, b, 2005, 2007, 2010, 2011, 2013, 2014; Kostanjšek and Celestina 2008; Kostanjšek and Fišer 2005; Kostanjšek and Gorjan 2013; Kostanjšek and Kuntner 2002; Kostanjšek and Miller 2004; Kostanjšek and Ramšak 2005; Kuntner 2001; Kuntner and Kostanjšek 2000; Kuntner et al. 2007; Neuhauser et al. 1995; Pipan et al. 2008; Polak et al. 2012), ecological and behavioral studies (Kralj-Fišer et al. 2013; Nentwig and M. 2010; Novak 2005b; Novak et al. 2004, 2010; Tkavc 2008) and zoogeographical reviews (Blick et al. 2004; Decae 2010; Deltshev 2005; 2008; Finch et al. 2008; Knapič 2012; Kuntner and Šereg 2002; Le Peru 2011, Řezáč et al. 2014).

## Materials and methods

The present checklist is based on all published literature containing information on spiders found in the territory of Slovenia but also includes several new records based on recently collected material. The classification and nomenclature follow the World Spider Catalog version 15.5 (2014). Families, genera and species are listed alphabetically. For each species, all relevant literature regarding its presence in Slovenia is followed by synonyms (as they occur in the literature) indicated by an equals sign (=). In this paper, all references relevant to Slovenian spider fauna are marked with [*] in order to distinguish them from other cited literature sources.

The new, previously unrecorded species in the list are marked by an asterisk and followed by a description of the collecting site, geographic data (latitude, longitude, altitude), date of collection and sampling method, and the data on the collector/material provider and species identifying authority. The specimens of previously unrecorded species are deposited in the collections of the Department of Biology, University of Ljubljana, Slovenia, and the Institute of Biology, Scientific Research Centre, Slovenian Academy of Sciences and Arts, Ljubljana, Slovenia.

### Species list

The Slovenian spider species list, published online as Araneae Sloveniae ver. 1.0 (http://www.bioportal.si/katalog/araneae.php), currently comprises 738 species belonging to 286 genera and 43 families (Table 1) and will be updated in due course. This list considerably exceeds the 590 spider species recorded to occur in Slovenia in Fauna Europaea (Van Helsdingen 2013) and almost doubles the records reported in the outdated checklist of Slovenian (within the Yugoslavian) spider fauna (Nikolić and Polenec 1981). This number was largely reached by adding original data published since the old catalogue but also by adding numerous overlooked records.

Numerous ambiguous records from the Yugoslav list (Nikolić and Polenec 1981) are clarified; these included 149 species and subspecies, marked by question marks, whose presence in Slovenia was expected but never confirmed. Although these dubious data were clearly marked as such in the Fauna Europaea Database (Van Helsdingen 2013), they continued to puzzle. For example, the widely used online identification key for European Spiders (Nentwig et al. 2014) marks the presence of both dubious and valid records on the Slovenia’s map. Of these dubious records, 75 species are retained in the checklist as they have recently been confirmed in the literature. The remaining 74 species and subspecies, however, are omitted. Together with other omissions from the list, such as nomina dubia, nomina nuda and redeterminations of published records, these are given at the end of the list.

**Table 1.** A taxonomic count of Slovenian spiders. See *Araneae Sloveniae* (http://www.bioportal.si/katalog/araneae.php) for updates.

Family	Genus	Species
Agelenidae	10	25
Amaurobiidae	2	8
Anapidae	1	1
Anyphaenidae	1	2
Araneidae	19	37
Atypidae	1	3
Clubionidae	1	20
Cybaeidae	2	4
Dictynidae	6	13
Dysderidae	7	21
Eresidae	1	1
Eutichuridae	1	9
Filistatidae	1	1
Gnaphosidae	16	53
Hahniidae	3	7
Leptonetidae	1	1
Linyphiidae	95	216
Liocranidae	5	9
Lycosidae	11	60
Mimetidae	1	3
Miturgidae	1	3
Mysmenidae	2	2
Nemesiidae	2	3
Nesticidae	2	4
Oecobiidae	2	2
Oxyopidae	1	3
Philodromidae	3	18
Pholcidae	4	5
Phrurolithidae	1	2
Pisauridae	2	3
Salticidae	29	66
Scytodidae	1	1
Segestriidae	1	5
Sparassidae	1	1
Tetragnathidae	4	13
Theridiidae	24	55
Theridiosomatidae	1	1
Thomisidae	13	43
Titanoecidae	2	4
Trachelidae	1	1
Uloboridae	2	3
Zodariidae	1	5
Zoropsidae	1	1
**43**	**286**	**738**

## Slovenian spider species checklist (alphabetically by families)

**Family AGELENIDAE**

***Agelena labyrinthica*** (Clerck, 1757)

Brignoli 1976, Čandek et al. 2013, Gregorič and Kuntner 2009, Kostanjšek 2004a, Kuntner 1996, 1997c, 1997d, 1999b, Kuntner and Kostanjšek 2000, Nikolić and Polenec 1981, Polenec 1964a, 1967b, 1970b, 1971a, 1971c, 1973e, 1974a, 1975b, 1978b, 1980a, 1989, Tarman 2003

= *Aranea roeselii* Scopoli, 1763

Scopoli 1763

***Allagelena gracilens*** (C. L. Koch, 1841)

= *Agelena gracilens* C. L. Koch, 1838

Brignoli 1976, Budja 2008, Čandek et al. 2013, Gregorič and Kuntner 2009, Kostanjšek 2004a, Kuntner 1996, 1997c, 1999b, Kuntner and Kostanjšek 2000, Nikolić and Polenec 1981, Polenec 1989

= *Agelena similis* Keyserling, 1863

Polenec 1957, 1958, 1973c, 1974a, 1978b

***Coelotes alpinus*** Polenec, 1972

Maurer 1982a, 1982b, Nikolić and Polenec 1981, Polenec 1972, 1973a, 1973b, 1973e, 1978b, 1982a, 1989, Tarman 2003, Thaler 1976, Zingerle 1999, Zupančič et al. 1984

***Coelotes atropos*** (Walckenaer, 1830)

Polenec 1959

***Coelotes poleneci*** Wiehle, 1964

Bole et al. 1980, Mršić 1997, Nikolić and Polenec 1981, Polenec 1961d, 1963d, 1964b, 1966a, 1966b, 1967a, 1967b, 1968a, 1969b, 1970a, 1970b, 1973b, 1973d, 1974b, 1975b, 1976a, 1976b, 1977, 1978c, 1979a, 1980b, 1982b, 1983, 1984, 1986, 1987, 1989, Tarman 2003, Thaler 1976, Wiehle 1964, Zupančič et al. 1984

***Coelotes solitarius*** L. Koch, 1868

Kostanjšek 2007, Novak 2005b, Thaler 1976

***Coelotes terrestris*** (Wider, 1834)

Čandek et al. 2013

***Eratigena agrestis*** (Walckenaer, 1802)

= *Tegenaria agrestis* (Walckenaer, 1802)

Brignoli 1976, Gregorič and Kuntner 2009, Nikolić and Polenec 1981, Polenec 1968b, 1984, Stražar 2001

***Eratigena atrica*** (C. L. Koch, 1843)

= *Tegenaria atrica* C. L. Koch, 1843

Čandek et al. 2013, Kostanjšek 2012, Polenec 1962b

***Hadites tegenarioides*** Keyserling, 1862

Joseph 1881, 1882

***Histopona luxurians*** (Kulczyński, 1897)

Deeleman-Reinhold 1983, Nikolić and Polenec 1981, Novak 2005b

= *Tegenaria luxurians* Kulczyński, 1897

Bole et al. 1980, 1982, Polenec 1960, 1961d, 1962a, 1963d, 1964a, 1965a, 1966a, 1966b, 1967a, 1967b, 1968a, 1969b, 1970b, 1971a, 1971c, 1973b, 1973d, 1974b, 1975b, 1976a, 1976b, 1977, 1978c, 1979a, 1980a, 1980b, 1982b, 1983, 1984, 1985, 1986, 1989, Wiehle 1964, Zupančič et al. 1984

***Histopona torpida*** (C. L. Koch, 1837)

Bole et al. 1980, Brignoli 1976, Budja 2008, Čandek et al. 2013, Gorjan 2014, Gregorič and Kuntner 2009, Kostanjšek 2004a, Kuntner 1996, 1997c, 1997d, 1999b, Kuntner and Baxter 1997, Kuntner and Kostanjšek 2000, Nikolić and Polenec 1981, Novak 1981, 2005b, Polak 1997, Polenec 1957, 1958, 1959, 1963e, 1964b, 1967b, 1970b, 1973e, 1974a, 1975a, 1975b, 1979a, 1984, 1985, 1986, 1987, 1989, Rebeušek et al. 2008

***Inermocoelotes anoplus*** (Kulczyński, 1897)

Čandek et al. 2013

= *Coelotes anoplus* Kulczyński, 1897

Bole et al. 1980, 1982, Brignoli 1975, 1976, Kuntner and Kostanjšek 2000, Nikolić and Polenec 1981, Polenec 1962a, 1963e, 1965b, 1966a, 1968a, 1968b, 1969a, 1973b, 1978d, 1979a, 1982b, 1985, 1989, Wiehle 1964

= *Eurocoelotes anoplus* (Kulczyński, 1897)

Wang et al. 2010

***Inermocoelotes inermis*** (L. Koch, 1855)

Gorjan 2014

= *Coelotes inermis* (L. Koch, 1855)

Bole et al. 1980, 1982, Brignoli 1976, Budja 2008, Kuntner 1997a, 1997c, 1999b, Nikolić and Polenec 1981, Novak 2005b, Polenec 1957, 1958, 1959, 1960, 1961d, 1962a, 1962c, 1963c, 1963d, 1964a, 1964b, 1965a, 1966a, 1966b, 1967a, 1967b, 1968a, 1969b, 1970a, 1970b, 1971a, 1971c, 1973c, 1973d, 1973e, 1974a, 1974b, 1975a, 1975b, 1976a, 1976b, 1977, 1978b, 1978c, 1979a, 1980a, 1981, 1982a, 1982b, 1983, 1984, 1985, 1986, 1987, 1989, Tarman 2003, Thaler 1976, Zupančič et al. 1984

= *Eurocoelotes inermis* (L. Koch, 1855)

Gregorič and Kuntner 2009, Wang et al. 2010

***Tegenaria campestris*** (C. L. Koch, 1834)

Kuntner 1999b, 2001, 2002, Polenec 1976a, ULRS 2002

***Tegenaria domestica*** (Clerck, 1757)

Kostanjšek 2004a, Kuntner 1999b, Nikolić and Polenec 1981, Polenec 1962b, 1978a, Tarman 2003

= *Tegenaria derhamii* Scopoli

Megušar 1914

***Tegenaria ferruginea*** (Panzer, 1804)

Kostanjšek 2004a, Nikolić and Polenec 1981, Novak 2005b, Polenec 1962b, 1989

Tegenaria pagana* (C. L. Koch, 1840)

1♀ - meadow, 2,5 km N from Kostanjevica na Krki; 45°52,5'N; 15°25,4'E, 150 m a. s. l.; 26.7.2009; leg. & det. Kostanjšek R.

***Tegenaria parietina*** (Fourcroy, 1785)

Kostanjšek 2004a, Nikolić and Polenec 1981, Novak 2005b, Polenec 1966b, 1989, Tarman 2003

***Tegenaria parvula*** Thorell, 1875

= *Tetrilus strandi* Caporiacco, 1936

Caporiacco 1938, Nikolić and Polenec 1981

***Tegenaria silvestris*** L. Koch, 1872

Bole et al. 1980, Brignoli 1976, 1980, Budja 2008, Kostanjšek 2003, 2004a, Kratochvíl 1934, Kratochvíl and Miller 1940, Kuntner 1997d, 1998, 1999b, Kuntner and Kostanjšek 2000, Nikolić and Polenec 1981, Novak 1981, 2005b, Novak and Kuštor 1982, Polenec 1957, 1958, 1959, 1960, 1963e, 1965b, 1967b, 1969a, 1969b, 1970a, 1970b, 1971c, 1974a, 1975a, 1975b, 1976b, 1978d, 1979a, 1980b, 1981, 1982a, 1984, 1985, 1986, 1987, 1989

= *Malthonica silvestris* (L. Koch, 1872)

Čandek et al. 2013, Gorjan 2014, Gregorič and Kuntner 2009

***Tegenaria tridentina*** L. Koch, 1872

Nikolić and Polenec 1981, Novak 2005b, Polenec 1964a, Wiehle 1964

***Textrix caudata*** L. Koch, 1872

Gregorič and Kuntner 2009, Nikolić and Polenec 1981, Polenec 1978d, 1992

***Textrix denticulata*** (Olivier, 1789)

Kostanjšek 2001

***Urocoras munieri*** (Simon, 1880)

= *Coelotes munieri* Simon, 1880

Bole et al. 1980, Nikolić and Polenec 1981

**Family AMAUROBIIDAE**

***Amaurobius erberi*** (Keyserling, 1863)

Bole et al. 1980, Čandek et al. 2013, Gorjan 2014, Kostanjšek 2002a, Polenec 1992

***Amaurobius fenestralis*** (Ström, 1768)

Bole et al. 1980, Fage 1931, Nikolić and Polenec 1981, Novak 2005b, Polenec 1979a, 1989, Tarman 2003

***Amaurobius ferox*** (Walckenaer, 1830)

Čandek et al. 2013, Gorjan 2014, Gregorič and Kuntner 2009, Kuntner 1999b, Kuntner and Kostanjšek 2000, Nikolić and Polenec 1981, Novak 2005b, Polenec 1989

***Amaurobius jugorum*** L. Koch, 1868

Budja 2008, Čandek et al. 2013, Kostanjšek 2004a, Nikolić and Polenec 1981, Polenec 1957, 1958, 1959, 1961d, 1963d, 1963e, 1964b, 1967b, 1968a, 1970b, 1971c, 1973c, 1974a, 1975a, 1975b, 1976b, 1985, 1989, Thaler 1990a

***Amaurobius minor*** (Kulczyński, 1915)

Nikolić and Polenec 1981, Polenec 1978d, 1979b, 1992

***Amaurobius obustus*** L. Koch, 1868

Bole et al. 1980, 1982, Nikolić and Polenec 1981, Polenec 1960, 1961d, 1962a, 1962c, 1963d, 1963e, 1964a, 1964b, 1965a, 1966a, 1966b, 1967a, 1967b, 1969a, 1969b, 1970a, 1970b, 1971a, 1973b, 1974b, 1975b, 1976a, 1976b, 1977, 1979a, 1980b, 1981, 1982a, 1982b, 1983, 1984, 1985, 1986, 1987, 1989, Thaler 1990a

***Amaurobius scopolii*** Thorell, 1871

Kratochvíl 1935

***Callobius claustrarius*** (Hahn, 1833)

Kostanjšek 2007, Nikolić and Polenec 1981

= *Amaurobius claustrarius* (Hahn, 1833)

Novak 2005b, Zupančič et al. 1984

**Family ANAPIDAE**

***Comaroma simoni*** Bertkau, 1889

Budja 2008, Gorjan 2014, Kostanjšek 2002b, Kuntner 1997c, Le Peru 2011, Nikolić and Polenec 1981, Polenec 1964b, 1966a, 1966b, 1970b, 1971a, 1971c, 1973b, 1983, 1989, Thaler 1978a

**Family ANYPHAENIDAE**

***Anyphaena accentuata*** (Walckenaer, 1802)

Budja 2008, Čandek et al. 2013, Kostanjšek 2013, Kuntner 1996, 1997c, 1997d, 1999b, Kuntner and Kostanjšek 2000, Nikolić and Polenec 1981, Novak 1981, 2005b, Polenec 1957, 1958, 1959, 1960, 1964b, 1973e, 1974a, 1985, 1986, 1987, 1989

***Anyphaena sabina*** L. Koch, 1866

Čandek et al. 2013

**Family ARANEIDAE**

***Aculepeira ceropegia*** (Walckenaer, 1802)

Čandek et al. 2013, Kuntner 1997d, Nikolić and Polenec 1981

= *Aranea ceropegia* Walckenaer, 1802

Polenec 1964b, 1971a, 1971c, 1973e, 1975b, 1976b

= *Araneus ceropegius* Lessert, 1910

Polenec 1989

***Agalenatea redii*** (Scopoli, 1763)

Čandek et al. 2013, Kostanjšek 2003

= *Aranea aldrovandi* Scopoli, 1763

Scopoli 1763

= *Aranea redii* Scopoli, 1763

Polenec 1954, Scopoli 1763

***Araneus alsine*** (Walckenaer, 1802)

Brigić et al. 2009, Čandek et al. 2013, Kostanjšek 2001, Kuntner 1997d, Kuntner and Kostanjšek 2000, Nikolić and Polenec 1981, Polak 1997, Polenec 1983, 1989

= *Aranea alsine* Walckenaer, 1802

Polenec 1957, 1958, 1973c, 1992

***Araneus angulatus*** Clerck, 1757

Čandek et al. 2013, Giltay 1932, Kostanjšek 2004a, 2005, Kuntner 1996, 1997a, 1997d, 1999b, 2002

***Araneus circe*** (Audouin, 1826)

Nikolić and Polenec 1981

= *Aranea circe* (Audouin, 1827)

Polenec 1992

***Araneus diadematus*** Clerck, 1757

Bedjanič 1999, Budja 2008, Čandek et al. 2013, Gregorič and Kuntner 2009, Kostanjšek 2004a, Kuntner 1996, 1997c, 1997d, 1999b, Kuntner and Kostanjšek 2000, Kuntner et al. 2008, Neuhauser et al. 1995, Nikolić 1963, Polak 1994, 1997, Polenec 1985, 1986, 1987, Tarman 2003, Vogrin 2002

= *Aranea duadema* Linnaeus, 1758

Polenec 1957, 1958, 1960, 1961b, 1964a, 1964b, 1965a, 1967b, 1969a, 1970b, 1971c, 1973c, 1974a, 1980a

= *Araneus diademus* Clerck, 1757

Polenec 1989

***Araneus marmoreus*** Clerck, 1757

Čandek et al. 2013, Kostanjšek 2004a, 2007, Kuntner 1997d, 1999b, Nikolić and Polenec 1981

= *Aranea raji* Scopoli, 1763

Polenec 1954, 1961b, 1980a, Scopoli 1763

= Aranea raji var. betulae Sulzer, 1776

Polenec 1961b, 1963d, 1964a, 1964b, 1965a, 1970b, 1989

= *Araneus marmoreus pyramidatus* Clerck, 1757

Kuntner 1997c, Nikolić and Polenec 1981

***Araneus quadratus*** Clerck, 1757

Čandek et al. 2013, Kuntner 1996, 1997c, 1997d, Kuntner and Kostanjšek 2000, Nikolić and Polenec 1981, Polenec 1989, Tarman 2003

= *Aranea quadrata* Clerck, 1757

Polenec 1978b

= *Aranea reaumiri* Scopoli, 1763

Polenec 1954

= *Aranea reaumurii* Scopoli, 1763

Scopoli 1763

***Araneus sturmi*** (Hahn, 1831)

Čandek et al. 2013, Kuntner 1997c, Kuntner and Kostanjšek 2000, Nikolić and Polenec 1981, Polenec 1989

= *Aranea sturmi* (Hahn, 1831)

Polenec 1957, 1958, 1960, 1973c

= *Atea sturmi* C.L.Koch, 1837

Budja 2008

***Araneus triguttatus*** (Fabricius, 1793)

Kostanjšek 2010

***Araniella alpica*** (L. Koch, 1869)

Kuntner et al. 2008

***Araniella cucurbitina*** (Clerck, 1757)

Čandek et al. 2013, 2015, Kostanjšek 2004a, 2005, Kuntner 1996, 1997c, 1997d, Nikolić and Polenec 1981, Novak 1981, Polenec 1984, Tarman 2003

= *Aranea cucurbitina* Linnaeus, 1758

Polenec 1963d, 1964a, 1967b, 1969a, 1969b, 1970b, 1971a, 1978b, 1980a

= *Aranea frischii* Scopoli, 1763

Scopoli 1763

= *Araneus cucurbitinus* Clerck, 1757

Novak 2005b, Polenec 1978d, 1985, 1989

***Araniella opistographa* (Kulczyński, 1905)**

Čandek et al. 2015

***Argiope bruennichi*** (Scopoli, 1772)

Bedjanič 1999, Čandek et al. 2013, Kaligarič 1996, Kostanjšek 2000, 2004a, 2004b, Kuntner 1996, 1997c, 1997d, 1999b, Kuntner and Kostanjšek 2000, Kuštor 2006, Nikolić and Polenec 1981, Polenec 1952, 1954, 1963b, 1966a, 1989, Tarman 2003, Vogrin 2002, 2005

= *Aranea brünnichii* Scopoli, 1772

Scopoli 1772

***Cercidia prominens*** (Westring, 1851)

Budja 2008, Čandek et al. 2013, Gregorič and Kuntner 2009, Kostanjšek 2003, Kuntner 1997d, 1999b, Kuntner and Kostanjšek 2000, Nikolić and Polenec 1981, Polenec 1957, 1958, 1973c, 1978d, 1986, 1989

***Cyclosa conica*** (Pallas, 1772)

Budja 2008, Čandek et al. 2013, Kostanjšek 2003, 2004a, Kuntner 1996, 1997c, 1997d, 1999b, Kuntner and Kostanjšek 2000, Kuntner et al. 2008, Nikolić and Polenec 1981, Polenec 1957, 1958, 1959, 1960, 1963d, 1964a, 1970b, 1973c, 1989

***Cyclosa oculata*** (Walckenaer, 1802)

Nikolić and Polenec 1981, Polenec 1957, 1958, 1964b, 1989

***Gibbaranea bituberculata*** (Walckenaer, 1802)

Budja 2008, Čandek et al. 2013, Kostanjšek 2003, Kuntner 2002

= *Aranea bituberculata* Walckenaer, 1802

Polenec 1969a

***Hypsosinga albovittata*** (Westring, 1851)

= *Singa albovittata* Westring, 1851

Polenec 1992

***Hypsosinga heri*** (Hahn, 1831)

Kostanjšek 2002b, 2014, Kuntner 1997c, 1999b, 2001, 2002, ULRS 2002

***Hypsosinga pygmaea*** (Sundevall, 1831)

Čandek et al. 2013, Kostanjšek 2004a, Kuntner 1996, 1999b

= *Singa pygmaea* (Sundevall, 1831)

Nikolić and Polenec 1981

***Hypsosinga sanguinea*** (C. L. Koch, 1844)

Čandek et al. 2013, Gorjan 2014, Kuntner 1997d

= *Singa sanguinea* C. L. Koch, 1844

Bole et al. 1980, Polenec 1989

***Larinioides cornutus*** (Clerck, 1757)

Kostanjšek 2004a, Kuntner 2002

= *Aranea cornuta* Clerck, 1757

Polenec 1992

= *Aranea leuwenhoekii* Scopoli, 1763

Scopoli 1763

***Larinioides sclopetarius*** (Clerck, 1757)

Čandek et al. 2013, Kostanjšek 2012, Kuntner 1997a, 1999b, 2002

***Larinioides suspicax*** (O. P.-Cambridge, 1876)

Kuntner 2002

***Leviellus thorelli*** (Ausserer, 1871)

Čandek et al. 2013, Gregorič 2008, Kralj-Fišer et al. 2013

= *Zygiella thorelli* (Ausserer, 1871)

Kostanjšek 2004a, Kuntner 1999b, 2002

***Mangora acalypha*** (Walckenaer, 1802)

Čandek et al. 2013, Kostanjšek 2003, 2004a, Kuntner 1997d, 1999b, Kuntner and Kostanjšek 2000, Nikolić and Polenec 1981, Polenec 1957, 1958, 1969a, 1969b, 1971a, 1973c, 1973e, 1989

***Neoscona adianta*** (Walckenaer, 1802)

Čandek et al. 2013, Kuntner 1997d, 2002, Kuntner and Kostanjšek 2000

= *Araneus adianta* Walckenaer, 1802

Giltay 1932

***Neoscona subfusca*** (C. L. Koch, 1837)

Kostanjšek and Kuntner 2002, Kuntner 2002, Kuntner and Kostanjšek 2000

***Nuctenea umbratica*** (Clerck, 1757)

Budja 2008, Čandek et al. 2013, Kuntner 1996, 1997d, 1999b, Kuntner and Baxter 1997, Kuntner and Kostanjšek 2000

= *Aranea sexpunctata* Linnaeus, 1758

Polenec 1962b, 1978b, 1980a

= *Aranea swammerdamii* Scopoli, 1763

Scopoli 1763

= *Araneus sexpunctatus* Linnaeus, 1758

Tarman 2003

= *Chinestela umbratica* (Clerck, 1757)

Nikolić and Polenec 1981

***Parazygiella montana*** (C. L. Koch, 1834)

Čandek et al. 2013, Gregorič 2008

= *Zilla montana* C. L. Koch, 1834

Polenec 1970a

= *Zygiella montana* (C. L. Koch, 1839)

Nikolić and Polenec 1981, Polenec 1979a, 1989

***Singa hamata*** (Clerck, 1757)

Kuntner 1997c, Nikolić and Polenec 1981

***Singa nitidula*** C. L. Koch, 1844

Čandek et al. 2013, Kuntner 1996, 1997c, 1999b, Nikolić and Polenec 1981

***Stroemiellus stroemi*** (Thorell, 1870)

Čandek et al. 2013, Gregorič 2008

= *Zygiella stroemi* (Thorell, 1870)

Gregorič 2008, Kuntner 1999b, 2001, 2002, ULRS 2002

***Zilla diodia*** (Walckenaer, 1802)

Čandek et al. 2013, Kostanjšek 2003, Kuntner 1996, 1997c, 1997d, Kuntner and Baxter 1997, Kuntner and Kostanjšek 2000, Nikolić and Polenec 1981

= *Aranea diodia* Walckenaer, 1802

Polenec 1958, 1969a

***Zygiella keyserlingi*** (Ausserer, 1871)

Gregorič 2008

***Zygiella x-notata*** (Clerck, 1757)

Gregorič 2008, Kuntner 2002, Kuntner and Kostanjšek 2000

= *Zilla litterata* Wiehle, 1931

Polenec 1962b

**Family ATYPIDAE**

***Atypus affinis*** Eichwald, 1830

Gorjan 2014, Gregorič and Kuntner 2009, Kostanjšek 2000, 2004a, Le Peru 2011, Nikolić and Polenec 1981

***Atypus muralis*** Bertkau, 1890

Bole et al. 1980, Kostanjšek 2000, 2001, Kuntner 1997d, Le Peru 2011, Nikolić and Polenec 1981, Polenec 1966a, 1969a

***Atypus piceus*** (Sulzer, 1776)

Čandek et al. 2013, Kostanjšek 2000, Kostanjšek and Kuntner 2002, Kuntner 1999b, Kuntner and Kostanjšek 2000, Le Peru 2011, Nikolić and Polenec 1981, Polenec 1957, 1958, 1961c, 1978a, 1978d, 1979b, Tarman 2003

= *Atypus picinus* 0

Bole et al. 1980

**Family CLUBIONIDAE**

***Clubiona brevipes*** Blackwall, 1841

Kuntner 1997d, Nikolić and Polenec 1981

***Clubiona caerulescens*** L. Koch, 1867

Bole et al. 1980, Budja 2008, Kuntner 1997d, 1999b, Nikolić and Polenec 1981, Polenec 1957, 1958, 1978d, 1979a, 1984, 1985, 1986, 1989

***Clubiona comta*** C. L. Koch, 1839

Gorjan 2014, Kuntner 1997d, 1999b, Nikolić and Polenec 1981, Polenec 1957, 1958, 1971a, 1978d

***Clubiona corticalis*** (Walckenaer, 1802)

Bole et al. 1982, Kuntner and Kostanjšek 2000, Polenec 1965b, 1992

= *Paraclubiona corticalis* (Walckenaer, 1802)

Nikolić and Polenec 1981

***Clubiona genevensis*** L. Koch, 1866

Polenec 1969a

= *Microclubiona genevensis* L. Koch, 1866

Nikolić and Polenec 1981

***Clubiona germanica*** Thorell, 1871

Čandek et al. 2013, Kuntner 1997c, 1999b, 2001, 2002, ULRS 2002

***Clubiona leucaspis*** Simon, 1932

Gorjan 2014, Kostanjšek and Gorjan 2014

***Clubiona lutescens*** Westring, 1851

Kuntner 1997c, 1997d, 1999b, Nikolić and Polenec 1981

***Clubiona marmorata*** L. Koch, 1866

Kuntner 1996, Kuntner and Baxter 1997, Polenec 1978d

= *Microclubiona marmorata* L. Koch, 1866

Nikolić and Polenec 1981

***Clubiona neglecta*** O. P.-Cambridge, 1862

Čandek et al. 2013, Nikolić and Polenec 1981, Polenec 1974b, 1989

***Clubiona pallidula*** (Clerck, 1757)

Bole et al. 1980, Kostanjšek 2004a, Nikolić and Polenec 1981, Novak 2005b, Polenec 1989

= *Clubiona holosericea* Linnaeus, 1758

Polenec 1969b

***Clubiona phragmitis*** C. L. Koch, 1843

Kuntner 1997d, 2001, 2002, ULRS 2002

***Clubiona pseudoneglecta*** Wunderlich, 1994

Čandek et al. 2013, Kostanjšek 2014, Kuntner 1997d, 2001, 2002, ULRS 2002

Clubiona reclusa* O. P.-Cambridge, 1863

1♂ - forest, Pokljuka, Zgornji Goreljek, 700 m W of Sivec peak; 46°20,05'N; 13°58,14'E, 1240 m a. s. l.; 1.11.2001; leg. & det. Kostanjšek R.

4♀ - forest, Pohorje, 500 m W from Lasina Peak, N of Rogla; 46°28,88'N; 15°20,24'E, 1300 m a. s. l.; 31.7.2005; leg. & det. Kostanjšek R.

4♀ - forest, Pohorje, Črne mlake marsh 1,6 km NE of Rogla; 46°28,12'N; 15°21,27'E, 1300 m a. s. l.; 31.7.2005; leg. & det. Kostanjšek R.

2♀ - damp meadow, 300 m E of Fara village near Kolpa river; 45°28,59'N; 14°53,18'E, 225 m a. s. l.; 23.7.2008; leg. & det. Kostanjšek R.

1♀ - meadow, Cerklje ob Krki; 150 m a. s. l.; 8.7.1981; det. Knapič T., Gregorič M.

1♀ - damp meadow, 3,6 km N of Kostanjevica na Krki; 45°52,73'N; 15°25,23'E, 150 m a. s. l.; 26.7.2009; leg. & det. Kostanjšek R.

***Clubiona similis*** L. Koch, 1867

Kostanjšek and Celestina 2008

***Clubiona stagnatilis*** Kulczyński, 1897

Kostanjšek 2000

***Clubiona subsultans*** Thorell, 1875

Nikolić and Polenec 1981, Polenec 1979a, 1989

***Clubiona subtilis*** L. Koch, 1867

Novak 1981, 2005b

***Clubiona terrestris*** Westring, 1851

Bole et al. 1980, Budja 2008, Čandek et al. 2013, Gorjan 2014, Gregorič and Kuntner 2009, Kuntner 1997d, Kuntner and Kostanjšek 2000, Nikolić and Polenec 1981, Novak 1981, 2005b, Polenec 1959, 1963e, 1969a, 1969b, 1973e, 1974a, 1978d, 1984, 1986, 1989

***Clubiona trivialis*** C. L. Koch, 1843

Polenec 1957, 1958, 1967b

= *Microclubiona trivialis* (C. L. Koch, 1843)

Nikolić and Polenec 1981

**Family CYBAEIDAE**

***Argyroneta aquatica*** (Clerck, 1757)

Budihna et al. 1996, Gregori 1980, 1994, Kalan et al. 2007, Kmecl 2000, Kostanjšek 2002a, 2004a, Kuntner 1997a, 2001, Nikolić and Polenec 1981, Polenec 1971a, 1992, Tarman 2003, ULRS 1993, 2002

***Cybaeus minor*** Chyzer, 1897

Bole et al. 1980, Maurer 1992, Nikolić and Polenec 1981, Polenec 1967a, 1968a, 1969b, 1970a, 1970b, 1971a, 1971c, 1973e, 1976a, 1977, 1978c, 1979a, 1980b, 1981, 1982b, 1983, 1984, 1985, 1986, 1989

***Cybaeus raymondi*** (Simon, 1916)

Kuntner 1998, 2001, Kuntner and Kostanjšek 2000, ULRS 2002

***Cybaeus tetricus*** (C. L. Koch, 1839)

Bole et al. 1980, Brignoli 1976, Kostanjšek 2004a, Maurer 1992, Nikolić and Polenec 1981, Novak 1981, 2005b, Polenec 1957, 1958, 1959, 1960, 1961d, 1962a, 1963d, 1964b, 1966a, 1967a, 1967b, 1968a, 1969a, 1969b, 1970a, 1970b, 1971c, 1973d, 1974a, 1974b, 1975a, 1975b, 1976a, 1976b, 1978c, 1979a, 1980a, 1980b, 1981, 1982b, 1983, 1985, 1989, Rebeušek et al. 2008, Zupančič et al. 1984

= *Cybaeus angustiarum* L. Koch, 1868

Novak 2005b

**Family DICTYNIDAE**

***Argenna subnigra*** (O. P.-Cambridge, 1861)

Čandek et al. 2013, Gorjan 2014, Gregorič and Kuntner 2009

***Cicurina cicur*** (Fabricius, 1793)

Bole et al. 1980, Brignoli 1976, Čandek et al. 2013, Novak 2005b, Polenec 1957, 1958, 1962a, 1963e, 1967b, 1974a, 1975a, 1976a, 1985, 1989, Roewer 1931

***Dictyna arundinacea*** (Linnaeus, 1758)

Nikolić and Polenec 1981, Polenec 1957, 1958

***Dictyna civica*** (Lucas, 1850)

Čandek et al. 2013, Kostanjšek 2012, Kostanjšek and Celestina 2008

***Dictyna latens*** (Fabricius, 1775)

Polenec 1971a

= *Brigittea latens* (Fabricius, 1775)

Nikolić and Polenec 1981

***Dictyna pusilla*** Thorell, 1856

Kuntner 1997c, 2002

***Dictyna uncinata*** Thorell, 1856

Čandek et al. 2013, Kuntner 1996, 1997a, 1997d, 2002

***Lathys humilis*** (Blackwall, 1855)

Čandek et al. 2013, 2015, Nikolić and Polenec 1981, Polenec 1978d

***Lathys nielseni*** (Schenkel, 1932)

Gregorič and Kuntner 2009

***Lathys stigmatisata*** (Menge, 1869)

Gorjan 2014, Kostanjšek and Gorjan 2014

***Mastigusa arietina*** (Thorell, 1871)

Gorjan 2014

= *Tetrilus arietinus* Thorell, 1881

Polenec 1981, 1989

= *Tetrilus macrophthalmus* Kulczyński, 1891

Novak 2005b, Polenec 1961d, 1963e, 1964a, 1966a, 1969a, 1975b, 1979a, 1989

***Mastigusa macrophthalma*** (Kulczyński, 1897)

Budja 2008, Kuntner and Kostanjšek 2000

= *Tuberta macrophthalma* (Kulczyński, 1897)

Nikolić and Polenec 1981

***Nigma walckenaeri*** (Roewer, 1951)

= *Dictyna viridissima* (Walckenaer, 1805)

Polenec 1988, 1989

**Family DYSDERIDAE**

***Dasumia canestrinii*** (L. Koch, 1876)

Bole et al. 1980, 1982, Budja 2008, Čandek et al. 2013, Gorjan 2014, Kuntner 1999b, Kuntner and Kostanjšek 2000, Le Peru 2011, Nikolić and Polenec 1981, Novak 1981, Polenec 1957, 1958, 1959, 1960, 1962a, 1963e, 1965a, 1965b, 1966a, 1966b, 1969a, 1973b, 1973c, 1976a, 1978d, 1979a, 1982b, 1984, 1985, 1986, 1989, Zupančič et al. 1984

= *Dasumia istriaca* Simon, 1882

Simon 1882

***Dasumia carpatica*** (Kulczyński, 1882)

= *Harpactea carpatica* (Kulczyński, 1882)

Polenec 1992

***Dysdera adriatica*** Kulczyński, 1897

Čandek et al. 2013, 2015, Gorjan 2014, Le Peru 2011, Polenec 1986, Řezáč et al. 2008a

= *Dysdera hungarica adriatica* Kulczyński, 1897

Nikolić and Polenec 1981

***Dysdera crocata*** C. L. Koch, 1838

Gregorič and Kuntner 2009

***Dysdera dubrovninnii*** Deeleman-Reinhold, 1988

Deeleman-Reinhold and Deeleman 1988, Le Peru 2011, Řezáč et al. 2008a

***Dysdera erythrina*** (Walckenaer, 1802)

Budja 2008,Gregorič and Kuntner 2009, Kostanjšek 2003, 2004a

***Dysdera hungarica*** Kulczyński, 1897

Budja 2008, Gorjan 2014, Polenec 1957, 1958, 1965a, 1983, 1984, 1985, 1989

***Dysdera kollari*** Doblika, 1853

Le Peru 2011

***Dysdera longirostris*** Doblika, 1853

Bole et al. 1980, Nikolić and Polenec 1981, Polenec 1961d, 1964b, 1969b, 1970a, 1970b, 1971c, 1974a, 1975a, 1975b, 1978d, 1979a, 1980b

***Dysdera microdonta*** Řezáč, 2014

Řezáč et al. 2014

***Dysdera ninnii*** Canestrini, 1868

Bole et al. 1980, 1982, Budja 2008, Čandek et al. 2013, Deeleman-Reinhold and Deeleman 1988, Gorjan 2014, Kostanjšek 2003, Kuntner 1996, 1998, Kuntner and Baxter 1997, Kuntner and Kostanjšek 2000, Kuntner et al. 1999b, Le Peru 2011, Nikolić and Polenec 1981, Polenec 1957, 1958, 1963d, 1963e, 1965a, 1965b, 1966a, 1967b, 1968a, 1968b, 1969a, 1973b, 1973c, 1974a, 1975a, 1975b, 1977, 1978d, 1982a, 1984, 1985, 1986, 1987, 1989, Řezáč et al. 2008a, 2014, Roewer 1931

***Harpactea grisea*** (Canestrini, 1868)

Alicata 1966, Bole et al. 1980, 1982, Le Peru 2011, Nikolić and Polenec 1981, Polenec 1980b, 1981, 1989, 1992, Thaler 1976

***Harpactea hombergi*** (Scopoli, 1763)

Budja 2008, Kuntner 1996, Kuntner and Baxter 1997, Le Peru 2011, Nikolić and Polenec 1981, Polenec 1989, 1992

= *Dysdera hombergi* Walckenaer, 1830

Damin 1900, Doblika 1853, Řezáč et al. 2008a

= *Harpactes hombergi* Scopoli, 1763

Polenec 1954, 1964b

***Harpactea lepida*** (C. L. Koch, 1838)

Bole et al. 1980, 1982, Budja 2008, Kuntner 1998, Kuntner et al. 1999b, Le Peru 2011, Nikolić and Polenec 1981, Novak 2005b, Polenec 1959, 1962c, 1966b, 1971a, 1977, 1978b, 1978c, 1979a, 1980a, 1980b, 1981, 1982a, 1982b, 1983, 1984, 1985, 1986, 1987, 1989, Thaler 1976, Zupančič et al. 1984

= *Harpactes lepidus* C. L. Koch, 1839

Polenec 1957, 1958, 1960, 1961a, 1961d, 1962a, 1962c, 1963d, 1963e, 1964a, 1964b, 1965a, 1966b, 1967a, 1967b, 1968a, 1969a, 1969b, 1970b, 1971a, 1971c, 1973c, 1973d, 1973e, 1974a, 1974b, 1975a, 1975b, 1976a, 1976b, 1978d

***Harpactea rubicunda*** (C. L. Koch, 1838)

Kostanjšek 2004a

***Mesostalita nocturna*** (Roewer, 1931)

Deeleman-Reinhold 1971a, Nikolić and Polenec 1981, Stepišnik and Ramšak 2006, ULRS 2002, 2004

= *Stalita nocturna* Roewer, 1931

Brignoli 1978, Caporiacco 1938, Gasparo 1996, Gasparo and Thaler 2000, Kratochvíl 1970, Le Peru 2011, Roewer 1931

***Parastalita stygia*** (Joseph, 1882)

Absolon and Kratochvíl 1933, Bedek et al. 2009, Caporiacco 1938, Deeleman-Reinhold 1971a, Kratochvíl 1934, 1970, Kržič 2010, Kuntner 1998, Kuntner et al. 1999b, Mršić 1997, Nikolić 1963, Nikolić and Polenec 1981, Polak 1994, Sket 2000, Tarman 2003, ULRS 2002

= *Stalita stygia* Joseph, 1882

Brignoli 1978, Drensky 1936, Fage 1931, Hamann 1896, Joseph 1882, Le Peru 2011, Wichmann 1926

***Rhode aspinifera*** (Nikolić, 1963)

Deeleman-Reinhold 1977a, 1993, Le Peru 2011, Nikolić and Polenec 1981, Tarman 2003, Thaler 1990b

= *Typhlorhode aspinifera* Nikolić, 1963

Nikolić 1961, 1963

***Stalita hadzii*** Kratochvíl, 1934

Brignoli 1971b, 1978, Kiauta and Leben 1960, Kratochvíl 1934, 1970, Le Peru 2011, Nikolić 1963, Nikolić and Polenec 1981, Polenec 1973b, Tarman 2003, ULRS 2002

***Stalita intermifemur*** Roewer, 1931

Caporiacco 1938, Kratochvíl 1970, Le Peru 2011, Nikolić and Polenec 1981, Roewer 1931

***Stalita taenaria*** Schiödte, 1847

Absolon and Kratochvíl 1933, Brignoli 1971b, 1978, 1980, Caporiacco 1938, Deeleman-Reinhold 1977a, Doleschall 1852, Drensky 1936, Fage 1931, Gasparo and Thaler 2000, Hafner 1925, Hamann 1896, Joseph 1881, 1882, Kostanjšek and Kuntner 2002, Kratochvíl 1934, 1970, Kržič 2010, Kuntner 1998, Kuntner and Kostanjšek 2000, Kuntner et al. 1999b, Kuštor and Novak 1980, Lämmermanr 1915, Le Peru 2011, Megušar 1914, Mršić 1997, Nikolić 1957, 1963, Novak 1976, Perko 1910, Pokorny 1853, Polak 1997, Polak et al. 2012, Polenec 1954, Pretner 1974, Reimoser 1919, Roewer 1931, Schiner 1854, Schiödte 1847, Sket 1979, 1993, 1999, 2000, 2006, Staut et al. 2008, Tarman 2003, ULRS 2002, Wichmann 1926

= *Stalita spinosissima* Kulczyński, 1897

Nikolić and Polenec 1981

**Family ERESIDAE**

***Eresus kollari*** Rossi, 1846

Řezáč et al. 2008b

= *Eresus cinnaberinus* (Olivier, 1789)

Gregorič and Kuntner 2009, Kuntner 1997d, Kuntner and Kostanjšek 2000, Nikolić and Polenec 1981, Tarman 2003

= *Eresus niger* Petagna, 1787

Kostanjšek and Kuntner 2002, Le Peru 2011, Polenec 1980a, 1989, 1992, ULRS 1993, 2002

**Family EUTICHURIDAE**

***Cheiracanthium elegans*** Thorell, 1875

Gregorič and Kuntner 2009, Nikolić and Polenec 1981

***Cheiracanthium erraticum*** (Walckenaer, 1802)

Čandek et al. 2013

= *Cheiracanthium dumetorum* (Hahn, 1833)

Nikolić and Polenec 1981, Tarman 2003

***Cheiracanthium mildei*** L. Koch, 1864

Čandek et al. 2013,Kostanjšek 2012, Kostanjšek and Celestina 2008

***Cheiracanthium montanum*** L. Koch, 1877

Polenec 1989

***Cheiracanthium pennyi*** O. P.-Cambridge, 1873

Kostanjšek 2002b

***Cheiracanthium punctorium*** (Villers, 1789)

Čandek et al. 2013, Gregorič and Kuntner 2009, Kostanjšek 2004a, 2013, Kuntner 1996, 1997d, Nikolić and Polenec 1981, Polenec 1964b, 1989, Tarman 2003

***Cheiracanthium rupestre*** Herman, 1879

Kratochvíl 1934, Nikolić and Polenec 1981

***Cheiracanthium striolatum*** Simon, 1878

Bole et al. 1980, Polenec 1992

***Cheiracanthium virescens*** (Sundevall, 1833)

Polenec 1989

**Family FILISTATIDAE**

***Filistata insidiatrix*** (Forsskål, 1775)

Čandek et al. 2013, Kuntner 1997d, 2001, 2002, Le Peru 2011, ULRS 2002

**Family GNAPHOSIDAE**

***Aphantaulax cincta*** (L. Koch, 1866)

Čandek et al. 2013, Gregorič and Kuntner 2009, Grimm 1985

***Aphantaulax trifasciata*** (O. P.-Cambridge, 1872)

Kostanjšek 2010

= *Aphantaulax seminigra* Simon, 1878

Grimm 1985

***Callilepis schuszteri*** (Herman, 1879)

Čandek et al. 2013, Gorjan 2014, Gregorič and Kuntner 2009, Grimm 1985, Kostanjšek 2005, Nikolić and Polenec 1981, Polenec 1978d

***Civizelotes pygmaeus*** (Miller, 1943)

= *Zelotes pygmaeus* (Miller, 1943)

Gregorič and Kuntner 2009

***Drassodes cupreus*** (Blackwall, 1834)

Bole et al. 1980, Kuntner 1997d, 2002

***Drassodes lapidosus*** (Walckenaer, 1802)

Bole et al. 1980, Čandek et al. 2013, Gorjan 2014, Gregorič and Kuntner 2009, Grimm 1985, Kostanjšek 2004a, Kuntner 1997d, 1999b, Kuntner and Kostanjšek 2000, Nikolić and Polenec 1981, Polenec 1968b, 1969a, 1970a, 1975a, 1978d, 1980b, 1981, 1982a, 1989

***Drassodes pubescens*** (Thorell, 1856)

Bole et al. 1980, Čandek et al. 2013, Gorjan 2014, Gregorič and Kuntner 2009, Grimm 1985, Kuntner 1997d, Kuntner and Kostanjšek 2000, Nikolić and Polenec 1981, Polenec 1957, 1958, 1963e, 1968b, 1969a, 1973c, 1975a, 1978d, 1980b, 1981, 1982a, 1985, 1989, Zupančič et al. 1984

***Drassyllus lutetianus*** (L. Koch, 1866)

Gorjan 2014, Kostanjšek 2004b

***Drassyllus praeficus*** (L. Koch, 1866)

Gorjan 2014, Gregorič and Kuntner 2009

= *Zelotes praeficus* (L. Koch, 1866)

Grimm 1985, Nikolić and Polenec 1981, Polenec 1957, 1958, 1968b, 1969a, 1973c, 1975a, 1976b, 1989

***Drassyllus pumilus*** (C. L. Koch, 1839)

Gorjan 2014, Gregorič and Kuntner 2009

***Drassyllus pusillus*** (C. L. Koch, 1833)

= *Zelotes pusillus* (C. L. Koch, 1833)

Grimm 1985, Nikolić and Polenec 1981, Polenec 1957, 1958, 1975b, 1989, 1992

***Drassyllus villicus*** (Thorell, 1875)

Čandek et al. 2013, Gorjan 2014, Gregorič and Kuntner 2009

= *Zelotes villicus* (Thorell, 1875)

Bole et al. 1980, Giltay 1932, Grimm 1985, Kuntner 1997d, Nikolić and Polenec 1981, Polenec 1969a, 1978d, 1983, 1986, 1989

***Echemus angustifrons*** (Westring, 1861)

Grimm 1985

= *Boreoechemus angustifrons* (Westring, 1861)

Polenec 1978d

= *Boreoechemus rhenanus* (Bertkau, 1883)

Nikolić and Polenec 1981

***Gnaphosa badia*** (L. Koch, 1866)

Bole et al. 1982, Grimm 1985, Nikolić and Polenec 1981, Polenec 1970a, 1971b, 1982a, 1989

***Gnaphosa bicolor*** (Hahn, 1833)

Bole et al. 1980, Čandek et al. 2013, Grimm 1985, Nikolić and Polenec 1981, Polenec 1957, 1958, 1973c, 1982a, 1985, 1989

***Gnaphosa inconspecta*** Simon, 1878

Nikolić and Polenec 1981, Polenec 1992

***Gnaphosa lucifuga*** (Walckenaer, 1802)

Grimm 1985, Nikolić and Polenec 1981, Polenec 1968b, 1969a, 1975a, 1992

***Gnaphosa lugubris*** (C. L. Koch, 1839)

Grimm 1985

***Gnaphosa modestior*** Kulczyński, 1897

Grimm 1985

***Gnaphosa muscorum*** (L. Koch, 1866)

Grimm 1985, Nikolić and Polenec 1981, Polenec 1969a, 1992

***Gnaphosa occidentalis*** Simon, 1878

= *Gnaphosa lugubris occidentalis* (Simon, 1873)

Nikolić and Polenec 1981

***Gnaphosa opaca*** Herman, 1879

Grimm 1985, Nikolić and Polenec 1981, Polenec 1978d, 1992

***Haplodrassus dalmatensis*** (L. Koch, 1866)

Bole et al. 1980, Gorjan 2014, Gregorič and Kuntner 2009, Grimm 1985, Polenec 1992

***Haplodrassus kulczynskii*** Lohmander, 1942

Grimm 1985, Nikolić and Polenec 1981, Polenec 1976b, 1989

***Haplodrassus signifer*** (C. L. Koch, 1839)

Gorjan 2014, Gregorič and Kuntner 2009, Grimm 1985, Nikolić and Polenec 1981, Polenec 1957, 1958, 1970b, 1989

= *Drassodes signifer* C. L. Koch, 1839

Bole et al. 1980, Polenec 1967b, 1975b

***Haplodrassus silvestris*** (Blackwall, 1833)

Bole et al. 1980, Čandek et al. 2013, Gregorič and Kuntner 2009, Grimm 1985, Kuntner 1997d, Nikolić and Polenec 1981, Polenec 1976b, 1978d, 1983, 1985, 1986, 1989

***Kishidaia conspicua*** (L. Koch, 1866)

= *Poecilochroa conspicua* (L. Koch, 1866)

Grimm 1985, Nikolić and Polenec 1981, Polenec 1978d, 1992

***Micaria aenea*** Thorell, 1871

Zupančič et al. 1984

***Micaria albovittata*** (Lucas, 1846)

Gregorič and Kuntner 2009

= *Micaria rogenhoferi* Herman, 1879

Nikolić and Polenec 1981, Polenec 1968b, 1975a

= *Micaria scintilans* (Cambridge, 1871)

Nikolić and Polenec 1981, Polenec 1969a

***Micaria dives*** (Lucas, 1846)

= *Micariolepis dives* (Lucas, 1846)

Nikolić and Polenec 1981, Polenec 1968b, 1975a, 1992

***Micaria formicaria*** (Sundevall, 1831)

Gregorič and Kuntner 2009, Kuntner 1997d, 2002

***Micaria fulgens*** (Walckenaer, 1802)

Bole et al. 1980, Gregorič and Kuntner 2009, Nikolić and Polenec 1981, Polenec 1957, 1958, 1963e, 1965a, 1973c, 1978d, 1981, 1985, 1989

***Micaria pulicaria*** (Sundevall, 1831)

Čandek et al. 2013, Polenec 1982a, 1989

***Micaria silesiaca*** L. Koch, 1875

Bole et al. 1980, Kostanjšek 2004b

***Nomisia aussereri*** (L. Koch, 1872)

Grimm 1985

***Nomisia exornata*** (C. L. Koch, 1839)

Čandek et al. 2013, Gorjan 2014, Grimm 1985, Kostanjšek 2010

***Phaeocedus braccatus*** (L. Koch, 1866)

Bole et al. 1982, Čandek et al. 2013, Polenec 1992

***Poecilochroa variana*** (C. L. Koch, 1839)

Gregorič and Kuntner 2009

***Scotophaeus scutulatus*** (L. Koch, 1866)

Čandek et al. 2013, Grimm 1985, Kostanjšek 2004a, 2005

***Trachyzelotes pedestris*** (C. L. Koch, 1837)

Čandek et al. 2013, Gregorič and Kuntner 2009

= *Zelotes pedestris* (C. L. Koch, 1837)

Bole et al. 1980, Grimm 1985, Nikolić and Polenec 1981, Polenec 1965b, 1968b, 1971c, 1975a, 1975b, 1977, 1978d, 1989

***Zelotes apricorum*** (L. Koch, 1876)

Bole et al. 1980, Čandek et al. 2013, Gregorič and Kuntner 2009, Grimm 1985, Nikolić and Polenec 1981, Polenec 1957, 1958, 1963e, 1965a, 1965b, 1968a, 1970a, 1970b, 1973c, 1978d, 1983, 1985, 1986, 1989

***Zelotes atrocaeruleus*** (Simon, 1878)

Gregorič and Kuntner 2009, Grimm 1985, Nikolić and Polenec 1981, Polenec 1966a, 1968b, 1969a, 1975a, 1983, 1989

***Zelotes clivicola*** (L. Koch, 1870)

Nikolić and Polenec 1981, Polenec 1968a, 1989

= *Zelotes clivicolus* Grimm, 1985

Grimm 1985

***Zelotes electus*** (C. L. Koch, 1839)

Grimm 1985, Nikolić and Polenec 1981, Polenec 1968b, 1969a, 1975a

***Zelotes erebeus*** (Thorell, 1871)

Bole et al. 1980, Gorjan 2014, Gregorič and Kuntner 2009, Grimm 1985, Nikolić and Polenec 1981, Polenec 1957, 1958, 1963e, 1965a, 1965b, 1966a, 1968b, 1969a, 1973b, 1973c, 1976a, 1978d, 1986, 1989

= *Zelotes serotinus* L. Koch, 1866

Polenec 1957, 1958, 1959, 1963d, 1965a, 1965b, 1966a, 1973c

***Zelotes exiguus*** (Müller & Schenkel, 1895)

Polenec 1992

***Zelotes gracilis*** (Canestrini, 1868)

Grimm 1985, Nikolić and Polenec 1981

***Zelotes hermani*** (Chyzer, 1897)

Gregorič and Kuntner 2009, Nikolić and Polenec 1981, Polenec 1968b, 1969a

***Zelotes latreillei*** (Simon, 1878)

Bole et al. 1980, Čandek et al. 2013, Grimm 1985, Kuntner 1997d, Nikolić and Polenec 1981, Polenec 1957, 1958, 1969a, 1969b, 1973c, 1975b, 1976b, 1977, 1978d, 1980b, 1984, 1986, 1989

***Zelotes longipes*** (L. Koch, 1866)

Gorjan 2014, Grimm 1985

***Zelotes oblongus*** (C. L. Koch, 1833)

Gorjan 2014, Gregorič and Kuntner 2009, Grimm 1985, Kuntner 2001, Polenec 1992, ULRS 2002

***Zelotes petrensis*** (C. L. Koch, 1839)

Bole et al. 1980, Gregorič and Kuntner 2009, Grimm 1985, Nikolić and Polenec 1981, Polenec 1957, 1958, 1965b, 1969a, 1973c, 1976b, 1979a, 1981, 1989

***Zelotes subterraneus*** (C. L. Koch, 1833)

Gorjan 2014, Nikolić and Polenec 1981, Polenec 1957, 1958, 1960, 1961d, 1963e, 1969a, 1973c, 1978d, 1986, 1989

**Family HAHNIIDAE**

***Antistea elegans*** (Blackwall, 1841)

Brignoli 1976, Nikolić and Polenec 1981, Polenec 1966b, 1967a, 1989

***Cryphoeca silvicola*** (C. L. Koch, 1834)

Brignoli 1976, Kratochvíl 1934, Nikolić and Polenec 1981, Polenec 1961d, 1967a, 1967b, 1970a, 1976a, 1979a, 1989, Zupančič et al. 1984

***Hahnia helveola*** Simon, 1875

Gregorič and Kuntner 2009, Polenec 1976b, 1979a, 1982a, 1985

= *Hahnia bressica* Simon, 1875

Nikolić and Polenec 1981, Polenec 1970b, 1974a, 1982a, 1989

***Hahnia montana*** (Blackwall, 1841)

Nikolić and Polenec 1981, Polenec 1970a, 1971b

Hahania nava* (Blackwall, 1841)

1♂ - forest, 3 km S of Rače in NE Slovenia; 46°25,73'N; 15°40,83'E, 250 m a. s. l.; 22.7.2013; leg. Velkavrh M., det.: Kuralt Ž., Kostanjšek R.

1♀ - forest, 1 km N of Marjeta na Dravskem polju, E of Rače in NE Slovenia; N 46°27,00'N, 15°43,32'E, 250 m a. s. l.; 18.7. - 24.7.2013; leg. Kostanjšek R., det.: Velkavrh M., Kostanjšek R.

***Hahnia ononidum*** Simon, 1875

Bole et al. 1980, Nikolić and Polenec 1981, Polenec 1978d, 1985, 1989

= *Hahnia mengei* Kulczyński, 1897

Polenec 1957, 1958, 1964b, 1973c

***Hahnia pusilla*** C. L. Koch, 1841

Budja 2008, Gorjan 2014, Kuntner 1997d, 1999b, Nikolić and Polenec 1981, Polenec 1957, 1958, 1959, 1960, 1962a, 1962c, 1964a, 1965a, 1965b, 1968b, 1970b, 1973c, 1973d, 1974a, 1974b, 1975a, 1975b, 1976a, 1977, 1989

**Family LEPTONETIDAE**

***Protoleptoneta italica*** (Simon, 1907)

Le Peru 2011

= *Paraleptoneta italica* Simon, 1917

Bole et al. 1982, Nikolić and Polenec 1981, Polenec 1973b, 1989

**Family LINYPHIIDAE**

Abacoproeces saltuum* (L. Koch, 1872)

1♂ - forest, 3 km S of Rače in NE Slovenia; 46°25,73'N, 15°40,83'E, 250 m a. s. l.; 22.7.2013; leg. Velkavrh M., det.: Kostanjšek R.

***Agnyphantes expunctus*** (O. P.-Cambridge, 1875)

Kostanjšek and Miller 2004

***Agyneta affinis*** (Kulczyński, 1898)

= *Aprolagus beatus* (Cambridge, 1906)

Nikolić and Polenec 1981

= *Meioneta beata* (O. P.-Cambridge, 1906)

Bole et al. 1980, Polenec 1971b

***Agyneta equestris*** (L. Koch, 1881)

= *Meioneta equestris* (L. Koch, 1881)

Nikolić and Polenec 1981, Polenec 1968b

***Agyneta fuscipalpa*** (C. L. Koch, 1836)

= *Meioneta fuscipalpa* (C. L. Koch, 1836)

Čandek et al. 2013

***Agyneta gulosa*** (L. Koch, 1869)

= *Meioneta gulosa* (L. Koch, 1869)

Nikolić and Polenec 1981, Polenec 1968a, 1970a, 1971b, 1989, 1992

***Agyneta innotabilis*** (O. P.-Cambridge, 1863)

= *Meioneta innotabilis* (O. P.-Cambridge, 1863)

Kostanjšek and Gorjan 2013

***Agyneta rurestris*** (C. L. Koch, 1836)

= *Meioneta rurestris* (C. L. Koch, 1836)

Bole et al. 1980, Čandek et al. 2013, Kostanjšek 2004a, Nikolić and Polenec 1981, Polenec 1967a, 1968a, 1970a, 1970b, 1971b, 1973b, 1978b, 1980a, 1989

***Agyneta saxatilis*** (Blackwall, 1844)

= *Aprolagus lugubris* (Blackwall, 1834)

Nikolić and Polenec 1981

= *Meioneta saxatilis* (Blackwall, 1844)

Bole et al. 1980, Čandek et al. 2013, Polenec 1957, 1958, 1973d, 1989, 1992

***Agyneta simplicitarsis*** (Simon, 1884)

= *Meioneta simplicitarsis* (Simon, 1884)

Čandek et al. 2013

***Anguliphantes monticola*** (Kulczyński, 1881)

= *Lepthyphantes monticola* (Kulczyński, 1882)

Nikolić and Polenec 1981, Novak 2005b, Polenec 1968a, 1970a, 1971b, 1973b, 1978b, 1979a, 1989, Tarman 2003, Zupančič et al. 1984

***Araeoncus anguineus*** (L. Koch, 1869)

Nikolić and Polenec 1981, Polenec 1970a, 1971b

Araeoncus humilis* (Blackwall, 1841)

1♂ - primary forest, Rajhenavski Rog sanctuary, Kočevje region; 45°41'N, 15°01'E, 870-920 m a. s. l.; 25-28.6.1999; leg. Floren A., det. Miller J.

***Asthenargus bracianus*** Miller, 1938

Kostanjšek and Gorjan 2013

***Bathyphantes gracilis*** (Blackwall, 1841)

Brignoli 1980, Kuntner 1997c, Nikolić and Polenec 1981, Novak 2005b, Polenec 1966b, 1967a, 1969b, 1975b, 1989

***Bathyphantes nigrinus*** (Westring, 1851)

Kuntner 1997c, 1999b, 2002, Polak 1997

***Bolyphantes alticeps*** (Sundevall, 1833)

Bole et al. 1980, Nikolić and Polenec 1981, Polenec 1967a, 1968a, 1970a, 1971b, 1973b, 1978b, 1978c, 1980a, 1981, 1982a, 1989, Zupančič et al. 1984

***Bolyphantes kolosvaryi*** (Caporiacco, 1936)

van Helsdingen et al. 2001

***Bolyphantes luteolus*** (Blackwall, 1833)

Bole et al. 1980, Nikolić and Polenec 1981, Polenec 1970a, 1971b, 1981, 1982a, 1989

***Centromerita bicolor*** (Blackwall, 1833)

Bole et al. 1980, Nikolić and Polenec 1981, Polenec 1967a, 1968a, 1970a, 1981, 1982a, 1989

***Centromerus capucinus*** (Simon, 1884)

Nikolić and Polenec 1981, Polenec 1962a, 1968b

***Centromerus cavernarum*** (L. Koch, 1872)

= *Centromerus jacksoni* Denis, 1952

Nikolić and Polenec 1981, Polenec 1967a, 1968a, 1969c, 1973d, 1976b, 1979a, 1982b, 1983, 1989

***Centromerus incilium*** (L. Koch, 1881)

Nikolić and Polenec 1981, Polenec 1962a, 1963e, 1965b, 1967b, 1969a, 1970a, 1975b, 1977, 1978d, 1979a, 1984, 1987, 1989

= *Centromerus incilius* L. Koch, 1881

Bole et al. 1980, Polenec 1961a, 1962a, 1962c, 1968b, 1969a, 1974b, 1975b, 1976a, 1981, 1989

***Centromerus leruthi*** Fage, 1933

Nikolić and Polenec 1981, Polenec 1978c, 1989

***Centromerus pabulator*** (O. P.-Cambridge, 1875)

Bole et al. 1980, Gorjan 2014, Nikolić and Polenec 1981, Polenec 1970a, 1979a, 1981, 1982a, 1982b, 1983, 1989, Zupančič et al. 1984

***Centromerus sellarius*** (Simon, 1884)

Bole et al. 1980, Gorjan 2014, Nikolić and Polenec 1981, Polenec 1961d, 1964a, 1964b, 1966b, 1967a, 1968a, 1969b, 1970a, 1970b, 1971c, 1974a, 1975a, 1976a, 1977, 1978c, 1979a, 1980b, 1983, 1989

***Centromerus serratus*** (O. P.-Cambridge, 1875)

Nikolić and Polenec 1981, Polenec 1978d, 1982a, 1985, 1989

***Centromerus silvicola*** (Kulczyński, 1887)

Bole et al. 1980, Gorjan 2014, Nikolić and Polenec 1981, Polenec 1975b, 1978c, 1979a, 1980b, 1981, 1982a, 1982b, 1983, 1986, 1987, 1989, Zupančič et al. 1984

= *Centromerus similis* Kulczyński, 1894

Polenec 1957, 1958, 1959, 1960, 1961a, 1961d, 1962a, 1962c, 1963e, 1964a, 1964b, 1966a, 1966b, 1967a, 1967b, 1968a, 1968b, 1969a, 1969b, 1970b, 1971a, 1971c, 1973b, 1973c, 1974a, 1974b, 1975a, 1975b, 1976a, 1976b, 1977

***Centromerus subalpinus*** Lessert, 1907

Nikolić and Polenec 1981, Polenec 1970a, 1971b, 1973b, Zupančič et al. 1984

***Centromerus sylvaticus*** (Blackwall, 1841)

Bole et al. 1980, Budja 2008, Gorjan 2014, Gregorič and Kuntner 2009, Nikolić and Polenec 1981, Novak 2005b, Polenec 1957, 1958, 1961a, 1962a, 1963e, 1969a, 1974b, 1975b, 1976a, 1978c, 1982a, 1984, 1989, Zupančič et al. 1984

***Centrophantes crosbyi*** (Fage & Kratochvíl, 1933)

Miller and Polenec 1975a, Nikolić and Polenec 1981, Polenec 1976b, 1978b, 1979a, 1980a, 1980b, 1983, 1984, 1987, 1989

= *Centromerus crosbyi* Fage & Kratochvíl, 1933

Polenec 1970b, 1971a, 1971c, 1973b, 1974b, 1989

***Centrophantes roeweri*** (Wiehle, 1961)

Bole et al. 1980, Komposch and Steinberger 1999, Nikolić and Polenec 1981, Novak 2005b, Polenec 1980a

= *Lepthyphantes roeweri* Wiehle, 1961

Carnelutti 1986, Nikolić and Polenec 1981, Polenec 1961d, 1966a, 1966b, 1967a, 1969c, Wiehle 1961

***Ceratinella brevipes*** (Westring, 1851)

Nikolić and Polenec 1981, Polenec 1957, 1959, 1967a, 1970a, 1977, 1979a, 1989

***Ceratinella brevis*** (Wider, 1834)

Bole et al. 1980, Nikolić and Polenec 1981, Polenec 1958, 1961d, 1965a, 1970a, 1973c, 1989

***Ceratinella major*** Kulczyński, 1894

Polenec 1963d, 1989

***Ceratinella scabrosa*** (O. P.-Cambridge, 1871)

Gorjan 2014, Nikolić and Polenec 1981, Polenec 1958, 1975b, 1978c, 1989

***Cinetata gradata*** (Simon, 1881)

Blick et al. 1995

= *Cineta gradata* (Simon, 1881)

Thaler 1972

***Cnephalocotes obscurus*** (Blackwall, 1834)

Kostanjšek 2014

***Collinsia inerrans*** (O. P.-Cambridge, 1885)

= *Milleriana inerrans* O. P.-Cambridge

Polenec 1970a, 1971b

***Cresmatoneta mutinensis*** (Canestrini, 1868)

Nikolić and Polenec 1981

***Dicymbium nigrum*** (Blackwall, 1834)

Bole et al. 1980, Nikolić and Polenec 1981, Polenec 1968a, 1989

***Diplocentria bidentata*** (Emerton, 1882)

Kuntner 2002, Kuntner and Kostanjšek 2000

***Diplocephalus alpinus*** (O. P.-Cambridge, 1872)

= *Diplocephalus conectens* Kulczyński, 1894

Novak 2005b, Polenec 1964b, 1967a, 1968a, 1970a, 1971a, 1989

= *Plaesiocraerus connectens* (Kulczyński, 1894)

Nikolić and Polenec 1981

***Diplocephalus crassilobus*** (Simon, 1884)

Čandek et al. 2013, Novak 2005b, Rebeušek et al. 2008

= *Diplocephalus crassiloba* Simon, 1884

Mildage 1979

***Diplocephalus cristatus*** (Blackwall, 1833)

Budja 2008, Kuntner 1997d, 2002, Novak 2005b

***Diplocephalus helleri*** (L. Koch, 1869)

Nikolić and Polenec 1981, Polenec 1970a, 1971b, 1992

***Diplocephalus latifrons*** (O. P.-Cambridge, 1863)

Kuntner 1998, Kuntner and Kostanjšek 2000, Nikolić and Polenec 1981, Novak 2005b, Polenec 1966b, 1976a, 1978c, 1979a, 1982a, 1989

***Diplocephalus picinus*** (Blackwall, 1841)

Bole et al. 1980, Nikolić and Polenec 1981, Polenec 1969b, 1976a, 1983, 1985, 1989

= *Savignia picina* Blackwell, 1841

Polenec 1957, 1958, 1964a

***Diplostyla concolor*** (Wider, 1834)

Čandek et al. 2013, Gorjan 2014, Kuntner 1997d, Kuntner and Kostanjšek 2000, Nikolić and Polenec 1981, Polenec 1986, 1989

= *Bathyphantes concolor* (Wider, 1834)

Bole et al. 1980, Polenec 1965b, 1974a, 1975a

= *Bolyphantes concolor* 0

Bole et al. 1980

= *Stylophora concolor* Wider

Polenec 1974b

***Dismodicus elevatus*** (C. L. Koch, 1838)

Nikolić and Polenec 1981, Polenec 1957, 1958

***Drapetisca socialis*** (Sundevall, 1833)

Budja 2008, Čandek et al. 2013, Kratochvíl 1934, Kuntner 1997d, 1999b, Nikolić and Polenec 1981, Novak 2005b, Polenec 1957, 1958, 1961d, 1962a, 1964a, 1964b, 1966b, 1967b, 1974a, 1975a, 1976b, 1977, 1979a, 1982a, 1985, 1987, 1989

= *Drapestica socialis* 0

Bole et al. 1980, Čandek et al. 2013

***Entelecara acuminata*** (Wider, 1834)

Čandek et al. 2013, Nikolić and Polenec 1981, Polenec 1957, 1958, 1982a, 1989

***Entelecara congenera*** (O. P.-Cambridge, 1879)

Polenec 1989

***Entelecara erythropus*** (Westring, 1851)

Polenec 1982a, 1989

***Entelecara media*** Kulczyński, 1887

Nikolić and Polenec 1981, Polenec 1970a, 1971b

***Erigone dentipalpis*** (Wider, 1834)

Gregorič and Kuntner 2009, Kuntner 1997d, Nikolić and Polenec 1981, Polenec 1967a, 1968a, 1970a, 1970b, 1971a, 1989

***Erigone longipalpis*** (Sundevall, 1830)

Polenec 1980b, 1989

***Erigonella hiemalis*** (Blackwall, 1841)

Nikolić and Polenec 1981, Polenec 1970a, 1989

***Erigonella subelevata*** (L. Koch, 1869)

Polenec 1971b, 1981, 1982a, 1989, Zupančič et al. 1984

***Floronia bucculenta*** (Clerck, 1757)

Čandek et al. 2013, Kuntner 1997c, 1997d, 1999b, Nikolić and Polenec 1981

= *Floronia frenata* (Wider, 1834)

Polenec 1957, 1958

***Formiphantes lephthyphantiformis*** (Strand, 1907)

= *Lepthyphantes lepthyphantiformis* (Strand, 1907)

Nikolić and Polenec 1981, Polenec 1978b, 1978c, 1979a, 1980a, 1989

= *Lepthyphantes pisai* Miller, 1951

Bole et al. 1980, Polenec 1967a, 1968a, 1969c, 1973b, 1974b

***Frontinellina frutetorum*** (C. L. Koch, 1834)

Čandek et al. 2013, Kuntner 1997d

= *Frontinella frutetorum* (C. L. Koch, 1834)

Čandek et al. 2013, Gregorič and Kuntner 2009, Kostanjšek 2003, Kuntner 1997d, Kuntner and Kostanjšek 2000, Nikolić and Polenec 1981

= *Linyphia frutetorum* C. L. Koch, 1834

Polenec 1969a, 1969b, 1971a, 1973d, 1989

= *Robertus frutetorum* 0

Bole et al. 1980

***Gnathonarium dentatum*** (Wider, 1834)

Polenec 1982a, 1989

***Gonatium hilare*** (Thorell, 1875)

Čandek et al. 2013, Nikolić and Polenec 1981, Polenec 1957, 1958, 1966a, 1973c, 1989

***Gonatium paradoxum*** (L. Koch, 1869)

= *Gonatium corallipes* (Cambridge, 1875)

Bole et al. 1980, Nikolić and Polenec 1981, Polenec 1965a, 1966b, 1974b, 1975b, 1978c, 1981, 1982a, 1983, 1984, 1985, 1986, 1989

***Gonatium rubellum*** (Blackwall, 1841)

Bole et al. 1980, Nikolić and Polenec 1981, Novak 2005b, Polenec 1964b, 1968a, 1969b, 1974b, 1978c, 1983, 1989, Thaler 1978a

= *Gonatium isabellinum* Menge, 1868

Polenec 1957, 1958, 1959, 1973c

***Gonatium rubens*** (Blackwall, 1833)

Bole et al. 1980, 1982, Gregorič and Kuntner 2009, Nikolić and Polenec 1981, Polenec 1977, 1978d, 1981, 1989

***Gongylidiellum edentatum*** Miller, 1951

Komposch and Steinberger 1999, Millidge 1979, Thaler 1973

***Gongylidium rufipes*** (Linnaeus, 1758)

Kostanjšek and Miller 2004

***Helophora insignis*** (Blackwall, 1841)

Kostanjšek 2010

***Hylyphantes nigritus*** (Simon, 1881)

Kostanjšek 2004a, Kostanjšek and Miller 2004

***Hypomma cornutum*** (Blackwall, 1833)

= *Enidia cornuta* (Blackwall, 1833)

Polenec 1988, 1989, 1992

***Hypsocephalus pusillus*** (Menge, 1869)

= *Cnephalocotes pusillus* Menge

Polenec 1957, 1958

***Improphantes decolor*** (Westring, 1861)

= *Lepthyphantes zebrinus* (Menge, 1866)

Bole et al. 1980

***Incestophantes annulatus*** (Kulczyński, 1882)

= *Lepthyphantes annulatus* Kulczyński, 1882

Budja 2008

***Ipa keyserlingi*** (Ausserer, 1867)

= *Centromerus keyserlingi* Ausserer, 1867

Polenec 1992

= *Lepthyphantes keyserlingi* (Ausserer, 1867)

Bole et al. 1980

***Kaestneria dorsalis*** (Wider, 1834)

Čandek et al. 2013, Kuntner 1999b, 2002

***Labulla thoracica*** (Wider, 1834)

Budja 2008, Kuntner 1997d, Nikolić and Polenec 1981, Novak 2005b, Polenec 1964b, 1970b, 1989

***Lepthyphantes leprosus*** (Ohlert, 1865)

Čandek et al. 2013, Nikolić and Polenec 1981, Polenec 1989

***Lepthyphantes minutus*** (Blackwall, 1833)

Čandek et al. 2013, Nikolić and Polenec 1981, Novak 2005b, Polenec 1979a, 1989, 1992

***Lepthyphantes notabilis*** Kulczyński, 1887

Novak 2005b

***Linyphia hortensis*** Sundevall, 1830

Čandek et al. 2013, Kostanjšek 2004a, Kuntner 1997d, 2002, Polenec 1978d

***Linyphia triangularis*** (Clerck, 1757)

Budja 2008, Čandek et al. 2013, Gregorič and Kuntner 2009, Kostanjšek 2004a, Kuntner 1996, 1997c, 1997d, 1999b, Kuntner and Baxter 1997, Kuntner and Kostanjšek 2000, Nikolić and Polenec 1981, Polenec 1961b, 1962a, 1963d, 1963e, 1967b, 1969a, 1969b, 1970b, 1971a, 1971c, 1973c, 1974a, 1974b, 1978b, 1980a, 1985, 1989, Tarman 2003

= *Aranea albini* (Scopoli, 1763)

Scopoli 1763

***Macrargus carpenteri*** (O. P.-Cambridge, 1894)

Polenec 1992

***Macrargus rufus*** Wider, 1834

Bole et al. 1980, 1982, Nikolić and Polenec 1981, Novak 2005b, Polenec 1957, 1958, 1959, 1961a, 1961d, 1962a, 1962c, 1963e, 1964a, 1964b, 1965a, 1965b, 1967a, 1967b, 1968a, 1969a, 1971c, 1973c, 1973d, 1973e, 1974b, 1975b, 1976a, 1976b, 1977, 1978d, 1982a, 1985, 1989

***Mansuphantes fragilis*** (Thorell, 1875)

= *Lepthyphantes fragilis* (Thorell, 1875)

Nikolić and Polenec 1981, Novak 2005b, Polenec 1961d, 1967a, 1968a, 1970a, 1971b, 1976a, 1979a, 1989, Wiehle 1961

***Mansuphantes mansuetus*** (Thorell, 1875)

Gorjan 2014, Gregorič and Kuntner 2009

= *Lepthyphantes mansuetus* (Thorell, 1875)

Bole et al. 1980, Nikolić and Polenec 1981, Polenec 1957, 1958, 1961a, 1961d, 1962a, 1963e, 1964b, 1965a, 1965b, 1966a, 1967a, 1967b, 1969a, 1969b, 1970b, 1973c, 1973e, 1974b, 1976a, 1978d, 1979a, 1981, 1982a, 1984, 1985, 1986, 1989, Thaler 1994, Zupančič et al. 1984

***Maso sundevalli*** (Westring, 1851)

Budja 2008, Čandek et al. 2013, Nikolić and Polenec 1981, Polenec 1957, 1958, 1961d, 1969b, 1989

***Mecopisthes silus*** (O. P.-Cambridge, 1872)

Nikolić and Polenec 1981, Polenec 1963d, 1967b, 1969a, 1970b, 1978d, 1980b, 1989

***Mecynargus foveatus*** (Dahl, 1912)

Čandek et al. 2013

***Megalepthyphantes collinus*** (L. Koch, 1872)

Čandek et al. 2013

***Megalepthyphantes nebulosus*** (Sundevall, 1830)

Gregorič and Kuntner 2009

= *Lepthyphantes nebulosa* (Sundevall, 1830)

Nikolić and Polenec 1981

***Mermessus trilobatus*** (Emerton, 1882)

Čandek et al. 2013

***Micrargus herbigradus*** (Blackwall, 1854)

Čandek et al. 2013, 2015, Kuntner 1999b, Nikolić and Polenec 1981, Polenec 1969a, 1971a, 1974a, 1975a, 1975b, 1976a, 1977, 1989

***Micrargus subaequalis*** (Westring, 1851)

= *Notocyba subaequalis* (Westring, 1851)

Zupančič et al. 1984

***Microlinyphia pusilla*** (Sundevall, 1830)

Kuntner 1997c, 1997d, 1999b, Nikolić and Polenec 1981, Polenec 1974b, 1984, 1989

= *Linyphia carnica* Caporiacco, 1922

Nikolić and Polenec 1981

= *Linyphia pusilla* (Sundevall, 1830)

Polenec 1957, 1958, 1973c

***Microneta viaria*** (Blackwall, 1841)

Bole et al. 1980, Kuntner 1999b, Nikolić and Polenec 1981, Polenec 1960, 1961d, 1962a, 1964a, 1964b, 1965b, 1966b, 1967a, 1968a, 1969b, 1970b, 1971a, 1971c, 1973d, 1974b, 1975b, 1976a, 1976b, 1978c, 1979a, 1983, 1984, 1986, 1989, Thaler 1978a

***Minicia marginella*** (Wider, 1834)

Čandek et al. 2013, Kuntner 1997d, 2002

***Minyriolus pusillus*** (Wider, 1834)

Nikolić and Polenec 1981, Polenec 1957, 1958, 1969a

***Moebelia penicillata*** (Westring, 1851)

Čandek et al. 2013, Polenec 1992

***Mughiphantes baebleri*** (Lessert, 1910)

= *Lepthyphantes baebleri* Lessert, 1910

Budja 2008

***Mughiphantes hadzii*** (Miller & Polenec, 1975)

= *Lepthyphantes hadzii* Miller & Polenec, 1975

Miller and Polenec 1975a, Nikolić and Polenec 1981, Tarman 2003

***Mughiphantes mughi*** (Fickert, 1875)

= *Lepthyphantes mughi* (Fickert, 1875)

Nikolić and Polenec 1981, Polenec 1970a, 1971b, Zupančič et al. 1984

***Mughiphantes triglavensis*** (Miller & Polenec, 1975)

= *Lepthyphantes triglavensis* Miller & Polenec, 1975

Komposch and Steinberger 1999, Miller and Polenec 1975a, Nikolić and Polenec 1981, Novak 2005b, Tarman 2003, Thaler 1976, 1990c

***Mughiphantes variabilis*** (Kulczyński, 1887)

= *Lepthyphantes variabilis* Kulczyński, 1887

Polenec 1971b

***Nematogmus sanguinolentus*** (Walckenaer, 1841)

Čandek et al. 2013, Kuntner and Kostanjšek 2000

***Neriene clathrata*** (Sundevall, 1830)

Čandek et al. 2013, 2015, Gorjan 2014, Gregorič and Kuntner 2009, Kuntner 1997d, Kuntner and Kostanjšek 2000, Nikolić and Polenec 1981, van Helsdingen 1969

= *Linyphia clathrata* Sundevall, 1829

Novak 2005b, Polenec 1957, 1958, 1963e, 1965a, 1965b, 1968b, 1969a, 1970b, 1975a, 1978b, 1978d, 1980a, 1989

***Neriene emphana*** (Walckenaer, 1841)

Budja 2008, Kostanjšek 2004a, Kuntner 1996, 1997c, 1997d, 1999b, Nikolić and Polenec 1981

= *Linyphia emphana* (Walckenaer, 1841)

Polenec 1974a, 1989

= *Prolinyphia emphana* Walckenaer, 1841

Polenec 1963d, 1964a, 1964b, 1970b, 1971a, 1971c

***Neriene furtiva*** (O. P.-Cambridge, 1871)

Čandek et al. 2013, Gorjan 2014, Gregorič and Kuntner 2009, Kuntner 1997d, 2002

***Neriene montana*** (Clerck, 1757)

Kuntner 1999b

= *Linyphia montana* (Clerck, 1757)

Polenec 1957, 1958, 1959, 1965a, 1968b, 1969a, 1978d

***Neriene peltata*** (Wider, 1834)

Budja 2008, Gorjan 2014, Kuntner 1997c, Nikolić and Polenec 1981, Novak 2005b, van Helsdingen 1969

= *Linyphia peltata* Wider, 1834

Novak 1981

= *Prolinyphia peltata* (Wider, 1834)

Polenec 1963d, 1964a, 1967b, 1969b, 1989

***Neriene radiata*** (Walckenaer, 1841)

Čandek et al. 2013, Kuntner 1997c, 1999b, van Helsdingen 1969

= *Linyphia marginata* C. L. Koch, 1834

Polenec 1957, 1958, 1965a, 1973c, 1989

= *Neriene marginata* C. L. Koch, 1834

Nikolić and Polenec 1981

= *Prolinyphia marginata* C. L. Koch, 1834

Polenec 1963d, 1964a, 1967b, 1970b

***Nusoncus nasutus*** (Schenkel, 1925)

= *Troxochrus nasutus* Schenkel, 1925

Polenec 1992, Zupančič et al. 1984

***Obscuriphantes obscurus*** (Blackwall, 1841)

= *Cnephalocotes obscurus* (Blackwall, 1834)

Kostanjšek 2010, Polenec 1981, 1982a, 1989

= *Lepthyphantes obscurus* (Blackwall, 1841)

Nikolić and Polenec 1981, Polenec 1957, 1958, 1960, 1969b, 1970b, 1989

***Oedothorax agrestis*** (Blackwall, 1853)

Nikolić and Polenec 1981, Polenec 1966b, 1967a, 1968a, 1974b, 1989

***Oedothorax apicatus*** (Blackwall, 1850)

Kostanjšek 2004a, Kostanjšek and Miller 2004

***Oedothorax retusus*** (Westring, 1851)

Kuntner 1997c, 2002

***Oreoneta montigena*** (L. Koch, 1872)

= *Hilaira montigena* (L. Koch, 1872)

Polenec 1970a, 1971b

***Oreonetides glacialis*** (L. Koch, 1872)

Polenec 1970a, 1971b

= *Montitetrix glacialis* (L. Koch, 1872)

Nikolić and Polenec 1981

***Oryphantes angulatus*** (O. P.-Cambridge, 1881)

Kuntner 2002

= *Lepthyphantes angulatus* O. P.-Cambridge, 1881

Kuntner and Kostanjšek 2000

***Ostearius melanopygius*** (O. P.-Cambridge, 1879)

Čandek et al. 2013

***Palliduphantes alutacius*** (Simon, 1884)

= *Lepthyphantes alutacius* Simon, 1884

Kuntner 1999b, 2002

***Palliduphantes istrianus*** (Kulczyński, 1914)

= *Lepthyphantes istrianus* Kulczyński, 1914

Bole et al. 1980, Nikolić and Polenec 1981, Polenec and Thaler 1980

***Palliduphantes pallidus*** (O. P.-Cambridge, 1871)

Gorjan 2014

= *Lepthyphantes pallidus* (O. P.-Cambridge, 1871)

Bole et al. 1980, Deeleman-Reinhold 1985, Nikolić and Polenec 1981, Novak 2005b, Polenec 1957, 1958, 1962a, 1963e, 1965b, 1969a, 1974a, 1975a, 1976a

= *Lepthyphantes pallidus alutatius* E. Simon, 1884 comb. nov. Miller, 1975

Polenec 1987, 1989, Zupančič et al. 1984

***Palliduphantes pillichi*** (Kulczyński, 1915)

= *Lepthyphantes pillichi* Kulczyński, 1915

Bole et al. 1980

***Panamomops affinis*** Miller & Kratochvíl, 1939

Bole et al. 1980, Polenec 1986, 1987, 1989

***Panamomops mengei*** Simon, 1926

Kostanjšek and Miller 2004

***Parapelecopsis nemoralis*** (Blackwall, 1841)

= *Pelecopsis nemoralis* (Blackwall, 1841)

Nikolić and Polenec 1981, Polenec 1957, 1958, 1973c, 1989

***Pelecopsis elongata*** (Wider, 1834)

Čandek et al. 2013, Nikolić and Polenec 1981, Polenec 1962a, 1962c, 1970a, 1989

***Pelecopsis radicicola*** (L. Koch, 1872)

Gorjan 2014, Nikolić and Polenec 1981, Polenec 1965a, 1975b, 1977, 1989

= *Pelecopsis thoracatus* Cambridge

Polenec 1957, 1958, 1973c

***Pityohyphantes phrygianus*** (C. L. Koch, 1836)

Nikolić and Polenec 1981, Polenec 1979a, 1989

***Pocadicnemis carpatica*** (Chyzer, 1894)

Kuntner 1999b, 2001, 2002, ULRS 2002

***Pocadicnemis pumila*** (Blackwall, 1841)

Čandek et al. 2013, Gorjan 2014, Polenec 1978d

Porrhomma campbelli* F. O. P.-Cambridge, 1894

1♀ - chasm Unška Koliševka, central Slovenia; 45°18,81'N, 14°15,81'E, 600 m a. s. l.; 1.9.2000 - 15.9.2001; leg. & det. Řezáč M., Kuntner M., Polak S.

***Porrhomma convexum*** (Westring, 1851)

Novak 1981, 2005b, Novak and Kuštor 1982, Polenec 1967a, 1973b, 1974b, 1989

= *Porrhomma proserpina* (Simon, 1872)

Kratochvíl 1934, Nikolić and Polenec 1981

***Porrhomma egeria*** Simon, 1884

Kiauta and Leben 1960, Kratochvíl 1934, Miller and Kratochvíl 1940, Novak et al. 1981, Polenec 1973b

***Porrhomma microphthalmum*** (O. P.-Cambridge, 1871)

Kostanjšek and Gorjan 2013

***Porrhomma microps*** (Roewer, 1931)

Novak 2005b

= *Porrhomma kolosvaryi* Kratochvíl, 1934

Kratochvíl 1934, Nikolić and Polenec 1981, Novak 2005b, Tarman 2003

***Porrhomma myops*** Simon, 1884

Novak 2005b

***Porrhomma pygmaeum*** (Blackwall, 1834)

Čandek et al. 2013, Kuntner 1997d, 1999b, Nikolić and Polenec 1981, Novak 2005b

***Porrhomma rosenhaueri*** (L. Koch, 1872)

Fage 1931, Nikolić and Polenec 1981, Polenec 1989, Tarman 2003

***Saaristoa firma*** (O. P.-Cambridge, 1905)

= *Oreonetides firmus* (O. P.-Cambridge, 1900)

Kratochvíl 1934, Nikolić and Polenec 1981, Novak 2005b, Polenec 1964b, 1969b, 1979a, 1989, Rebeušek et al. 2008, Thaler 1981b

***Saloca diceros*** (O. P.-Cambridge, 1871)

Gorjan 2014, Nikolić and Polenec 1981, Polenec 1963d, 1968a, 1973d, 1974b, 1979a, 1989

***Sauron rayi*** (Simon, 1881)

Gorjan 2014

= *Metopobactrus rayi* (Simon, 1881)

Nikolić and Polenec 1981, Polenec 1978d

***Scotargus pilosus*** Simon, 1913

Bole et al. 1980, Nikolić and Polenec 1981, Polenec 1968a, 1969c, 1970a, 1974a, 1974b, 1977, 1978c, 1979a, 1980b, 1989

= *Macrargus strandi* (Schenkel, 1934)

Polenec 1966b, 1967a, 1967b, 1969c

***Sintula corniger*** (Blackwall, 1856)

Bole et al. 1980, Gorjan 2014, Polenec 1970a, 1976a

***Sintula retroversus*** (O. P.-Cambridge, 1875)

Gorjan 2014, Nikolić and Polenec 1981

***Sintula spiniger*** (Balogh, 1935)

Gorjan 2014, Gregorič and Kuntner 2009

= *Sintula spininigra* (Balogh, 1935)

Bole et al. 1982

***Stemonyphantes conspersus*** (L. Koch, 1879)

= *Stemonyphantes pictus* Buchar, 1967

Polenec 1992

***Stemonyphantes lineatus*** (Linnaeus, 1758)

Bole et al. 1980, Gorjan 2014, Gregorič and Kuntner 2009, Nikolić and Polenec 1981, Polenec 1957, 1958, 1961a, 1961d, 1962a, 1963e, 1965b, 1966a, 1967b, 1968a, 1968b, 1969a, 1970a, 1973c, 1974b, 1975b, 1976a, 1989, Zupančič et al. 1984

***Styloctetor stativus*** (Simon, 1881)

Gorjan 2014

= *Anacotyle stativa* Simon, 1926

Bole et al. 1982

***Syedra gracilis*** (Menge, 1869)

Bole et al. 1980, Polenec 1968b, 1975a

***Tallusia experta*** (O. P.-Cambridge, 1871)

Gorjan 2014

= *Centromerus expertus* (O. P.-Cambridge, 1871)

Bole et al. 1980, Polenec 1992

***Tallusia vindobonensis*** (Kulczyński, 1898)

Thaler 1997

= *Centromerus vindobonensis* Kulczyński, 1898

Polenec and Thaler 1980

***Tapinocyba insecta*** (L. Koch, 1869)

Kuntner 1999b, Polenec 1976b, 1984, 1989

***Tapinocyba pallens*** (O. P.-Cambridge, 1872)

Nikolić and Polenec 1981, Polenec 1957, 1958, 1959, 1960, 1961d, 1963e, 1965a, 1965b, 1967b, 1969a, 1970a, 1971a, 1973c, 1973e, 1976a, 1989

***Tapinopa longidens*** (Wider, 1834)

Bole et al. 1980, Gorjan 2014, Gregorič and Kuntner 2009, Nikolić and Polenec 1981, Polenec 1957, 1958, 1961d, 1963e, 1968a, 1968b, 1969a, 1970a, 1970b, 1978b, 1989

***Tenuiphantes alacris*** (Blackwall, 1853)

= *Lepthyphantes alacris* (Blackwall, 1853)

Bole et al. 1980, Kuntner 1998, Kuntner and Kostanjšek 2000, Nikolić and Polenec 1981, Novak 2005b, Polenec 1961d, 1967a, 1968a, 1970a, 1970b, 1973e, 1974b, 1978c, 1979a, 1989, Zupančič et al. 1984

***Tenuiphantes cristatus*** (Menge, 1866)

Gorjan 2014, Gregorič and Kuntner 2009

= *Lepthyphantes cristatus* (Menge, 1866)

Bole et al. 1980, Nikolić and Polenec 1981, Novak 1981, 2005b, Novak and Kuštor 1982, Polenec 1957, 1958, 1959, 1960, 1961a, 1961d, 1962a, 1962c, 1963e, 1964a, 1964b, 1965a, 1966a, 1967a, 1967b, 1968a, 1969a, 1970a, 1971c, 1973c, 1973e, 1974a, 1974b, 1975a, 1976a, 1977, 1978c, 1979a, 1983, 1985, 1986, 1987, 1989, Zupančič et al. 1984

***Tenuiphantes flavipes*** (Blackwall, 1854)

Čandek et al. 2013, Gorjan 2014

= *Lepthyphantes flavipes* (Blackwall, 1854)

Bole et al. 1980, Budja 2008, Kuntner 1997d, 1999b, Kuntner and Kostanjšek 2000, Nikolić and Polenec 1981, Novak 2005b, Polenec 1957, 1958, 1959, 1962a, 1963e, 1964a, 1965a, 1965b, 1967b, 1969a, 1970a, 1970b, 1973e, 1974a, 1974b, 1975a, 1975b, 1976b, 1977, 1978d, 1979a, 1982a, 1985, 1989, Thaler 1978a

***Tenuiphantes mengei*** (Kulczyński, 1887)

Gorjan 2014

= *Lepthyphantes mengei* Kulczyński, 1887

Bole et al. 1980, Budja 2008, Kuntner and Kostanjšek 2000, Polenec 1957, 1958, 1961a, 1962a, 1963e, 1965b, 1968b, 1973c, 1976a, 1981, 1989

***Tenuiphantes tenebricola*** (Wider, 1834)

Gorjan 2014, Kostanjšek 2004a

= *Lepthyphantes pygmaeus* (Menge, 1866)

Nikolić and Polenec 1981

= *Lepthyphantes tenebricola* (Wider, 1834)

Bole et al. 1980, 1982, Budja 2008, Kuntner 1997c, 1999b, Kuntner and Kostanjšek 2000, Nikolić and Polenec 1981, Novak 2005b, Polenec 1957, 1958, 1959, 1960, 1961d, 1962a, 1963d, 1963e, 1964a, 1966b, 1967a, 1967b, 1968a, 1969a, 1969b, 1970a, 1970b, 1971a, 1971c, 1973d, 1973e, 1974a, 1974b, 1975a, 1975b, 1976a, 1976b, 1977, 1978b, 1978c, 1979a, 1980b, 1983, 1984, 1986, 1987, 1989, Zupančič et al. 1984

***Tenuiphantes tenuis*** (Blackwall, 1852)

Čandek et al. 2013, Gorjan 2014, Gregorič and Kuntner 2009

***Tenuiphantes zimmermanni*** (Bertkau, 1890)

Gorjan 2014, Kostanjšek 2004a

= *Bathyphantes simonii* Bösenberg, 1901

Nikolić 1963

= *Lepthyphantes zimmermanni* Bertkau, 1890

Kuntner and Kostanjšek 2000, Nikolić and Polenec 1981, Polenec 1957, 1958, 1971c, 1976b, 1977, 1989

***Theonina cornix*** (Simon, 1881)

Čandek et al. 2013, Gorjan 2014, Nikolić and Polenec 1981, Polenec 1978d

***Thyreostenius parasiticus*** (Westring, 1851)

Polenec 1987, 1989, Tarman 2003

***Tiso aestivus*** (L. Koch, 1872)

Nikolić and Polenec 1981, Polenec 1970a, 1971b, Tarman 2003

***Tiso vagans*** (Blackwall, 1834)

Nikolić and Polenec 1981, Polenec 1970a, 1971b, 1978c, 1980b, 1982a, 1989

***Trematocephalus cristatus*** (Wider, 1834)

Kuntner 1997c, 1997d, Polenec 1992

***Trichoncus affinis*** Kulczyński, 1894

Gorjan 2014, Nikolić and Polenec 1981, Polenec 1963e, 1986, 1989

***Trichoncus hackmani*** Millidge, 1955

Gorjan 2014, Gregorič and Kuntner 2009, Kostanjšek 2004a, Nikolić and Polenec 1981, Polenec 1978d

= *Trichoncus vasconicus hackmani* Millidge

Polenec 1971a

***Trichoncus saxicola*** (O. P.-Cambridge, 1861)

Kostanjšek and Gorjan 2013

***Trichoncus scrofa*** Simon, 1884

Polenec 1969a

***Trichoncyboides simoni*** (Lessert, 1904)

= *Tapinocyboides simoni* (Lessert, 1904)

Polenec 1992

***Trichopterna cito*** (O. P.-Cambridge, 1872)

Nikolić and Polenec 1981

***Troglohyphantes brignolii*** Deeleman-Reinhold, 1978

Polak et al. 2012

***Troglohyphantes confusus*** Kratochvíl, 1939

Bole et al. 1993, Deeleman-Reinhold 1973b, 1978, Kratochvíl 1939, Kuntner 2001, Nikolić and Polenec 1981, Polenec 1977, 1979a, 1983, 1986, 1989, 1992, Růžička et al. 2005, Sket 1979, Tarman 2003, Thaler 1986, ULRS 2002

***Troglohyphantes croaticus*** (Chyzer, 1894)

Kratochvíl 1939

***Troglohyphantes cruentus*** Brignoli, 1971

Brignoli 1971b, Deeleman-Reinhold 1978, Nikolić and Polenec 1981, ULRS 2002

***Troglohyphantes diabolicus*** Deeleman-Reinhold, 1978

Batič et al. 1980, Bole et al. 1993, Deeleman-Reinhold 1978, Kuntner 2001, Mršić 1997, Nikolić and Polenec 1981, Novak 1981, 2005b, Novak and Kuštor 1982, Pipan et al. 2008, Polenec 1992, Puc et al. 1988, Rebeušek et al. 2008, ULRS 2002

***Troglohyphantes diurnus*** Kratochvíl, 1932

Deeleman-Reinhold 1973b, Drensky 1936, Kratochvíl 1934, 1939, 1948, Nikolić and Polenec 1981, Novak 2005b, Thaler and Polenec 1974

***Troglohyphantes excavatus*** Fage, 1919

Bole et al. 1980, Brignoli 1971b, 1980, Deeleman-Reinhold 1971b, 1973a, 1978, Fage 1919, 1931, Gasparo and Thaler 2000, Kratochvíl 1934, Nikolić 1963, Nikolić and Polenec 1981, Novak 2005b, Pesarini 1989, Polak et al. 2012, Polenec 1963d, 1966a, 1967a, 1969b, 1969c, 1971a, 1973b, 1977, 1978c, 1979a, 1982b, 1983, 1985, 1986, 1989, Rebeušek et al. 2008, Roewer 1931, Thaler 1986, Wiehle 1964, Zupančič et al. 1984

= *Troglohyphantes anellii* Capoiracco, 1938

Brignoli 1971b, Caporiacco 1938

***Troglohyphantes fagei*** Roewer, 1931

Nikolić and Polenec 1981

***Troglohyphantes gamsi*** Deeleman-Reinhold, 1978

Deeleman-Reinhold 1978, Nikolić and Polenec 1981, Novak 2005b, Tarman 2003, ULRS 2002

***Troglohyphantes gracilis*** Fage, 1919

Deeleman-Reinhold 1978, Drensky 1936, Fage 1919, 1931, Kratochvíl 1934, Nikolić and Polenec 1981, ULRS 2002

***Troglohyphantes helsdingeni*** Deeleman-Reinhold, 1978

Deeleman-Reinhold 1978, Kuntner 2001, Nikolić and Polenec 1981, Novak 2005b, Polenec 1992, Thaler 1986, ULRS 2002

***Troglohyphantes jamatus*** Roewer, 1931

Deeleman-Reinhold 1978, Nikolić and Polenec 1981, Roewer 1931, Sket 1979, 2000, Tarman 2003, ULRS 2002

= *Troglohyphantes jugoslavicus* Kratochvíl, 1934

Drensky 1936, Kratochvíl 1934, 1939

***Troglohyphantes karawankorum*** Deeleman-Reinhold, 1978

Deeleman-Reinhold 1978, Nikolić and Polenec 1981, Novak 2005b, Tarman 2003, Thaler 1986, ULRS 2002

***Troglohyphantes latzeli*** Thaler, 1986

Thaler 1986

***Troglohyphantes novicordis*** Thaler, 1978

Novak 2005b

***Troglohyphantes poleneci*** Wiehle, 1964

Carnelutti 1986, Deeleman-Reinhold 1973b, 1978, Kuntner 2001, Miller and Polenec 1975b, Nikolić and Polenec 1981, Novak 2005b, Polenec 1966a, 1966b, 1967a, 1969b, 1969c, 1970b, 1973d, 1974b, 1975b, 1976a, 1976b, 1980a, 1983, 1984, 1986, 1987, 1989, 1992, Tarman 2003, ULRS 2002, Wiehle 1964

= *Troglohyphantes montanus* Kratochvíl, 1934

Polenec 1970a, 1971c, 1973b

***Troglohyphantes polyophthalmus*** Joseph, 1881

Brignoli 1971b, Deeleman-Reinhold 1971b, 1973b, 1975, 1978, Drensky 1936, Drovenik 1971, Fage 1919, Hamann 1896, Joseph 1881, 1882, Kratochvíl 1934, Nikolić and Polenec 1981, Novak 2005b, Novak et al. 2004, Pavlek and Ozimec 2009, Polenec 1973b, 1979a, 1989, Reimoser 1919, Stražar 2001, ULRS 2002, Zupančič et al. 1984

= *Nicthyphantes microphthalmus* Joseph, 1881

Hamann 1896, Joseph 1881, 1882

***Troglohyphantes sbordonii*** Brignoli, 1975

Gasparo 2000, Novak 2005b

= *Troglohyphantes cornutus* Deeleman-Reinhold, 1978

Deeleman-Reinhold 1978, Nikolić and Polenec 1981, Novak 2005b, Thaler 1982, ULRS 2002

***Troglohyphantes scientificus*** Deeleman-Reinhold, 1978

Deeleman-Reinhold 1978, ULRS 2002

***Troglohyphantes similis*** Fage, 1919

Deeleman-Reinhold 1978, Drensky 1936, Fage 1919, 1931, Nikolić and Polenec 1981, Tarman 2003, ULRS 2002

= *Troglohyphantes similis dubius* Kratochvíl, 1934

Kratochvíl 1934

***Troglohyphantes sketi*** Deeleman-Reinhold, 1978

Deeleman-Reinhold 1978, Mršić 1997, Nikolić and Polenec 1981, Pavlek and Ozimec 2009, Tarman 2003, ULRS 2002

***Troglohyphantes sordellii*** (Pavesi, 1875)

= *Troglohyphantes ghidinii* (Lessert, 1906)

Nikolić 1963

***Troglohyphantes spinipes*** Fage, 1919

Deeleman-Reinhold 1978, Drensky 1936, Fage 1919, 1931, Nikolić and Polenec 1981, ULRS 2002

= *Troglohyphantes similis spinipes* Fage, 1919

Kratochvíl 1934

***Troglohyphantes subalpinus*** Thaler, 1967

Novak 2005b, Pavlek and Ozimec 2009

***Troglohyphantes thaleri*** Miller & Polenec, 1975

Deeleman-Reinhold 1978, Kuntner 2001, Miller and Polenec 1975b, Nikolić and Polenec 1981, Novak 2005b, Polenec 1992, Thaler 1978b, ULRS 2002

***Troglohyphantes trispinosus*** Miller & Polenec, 1975

Deeleman-Reinhold 1978, Kuntner 2001, Miller and Polenec 1975b, Nikolić and Polenec 1981, Polenec 1978c, 1979a, 1980b, 1981, 1982b, 1983, 1985, 1989, 1992, Tarman 2003, ULRS 2002

***Troglohyphantes typhlonetiformis*** Absolon & Kratochvíl, 1932

Absolon and Kratochvíl 1932, Deeleman-Reinhold 1973a, 1978, Kiauta and Leben 1960, Nikolić and Polenec 1981, Novak 2005b, Polenec 1973b, Tarman 2003, ULRS 2002

***Troglohyphantes vicinus*** Miller & Polenec, 1975

Deeleman-Reinhold 1978, Kuntner 2001, Miller and Polenec 1975b, Nikolić and Polenec 1981, Polenec 1978b, 1983, 1989, 1992, ULRS 2002

***Troglohyphantes wiehlei*** Miller & Polenec, 1975

Deeleman-Reinhold 1978, Komposch and Steinberger 1999, Kuntner 2001, Miller and Polenec 1975b, Nikolić and Polenec 1981, Novak 2005b, Polenec 1983, 1986, 1989, 1992, Thaler 1986, ULRS 2002

***Walckenaeria acuminata*** Blackwall, 1833

Polenec 1974a

= *Wideria acuminata* 0

Bole et al. 1980

***Walckenaeria antica*** (Wider, 1834)

Gorjan 2014, Polenec 1957, 1958, 1973c

= *Wideria antica* (Wider, 1834)

Bole et al. 1980, Nikolić and Polenec 1981, Polenec 1963e, 1965a, 1965b, 1968b, 1969a, 1975b, 1981, 1986, 1989

***Walckenaeria atrotibialis*** (O. P.-Cambridge, 1878)

Gorjan 2014

= *Walckenaeria melanocephala* Cambridge, 1881

Polenec 1957, 1958, 1973c

= *Wideria atrotibialis* (Cambridge, 1878)

Nikolić and Polenec 1981

= *Wideria melanocephala* Cambridge, 1881

Bole et al. 1980, Polenec 1964b, 1965a, 1967b, 1969a, 1971a, 1986, 1989

***Walckenaeria capito*** (Westring, 1861)

= *Wideria capito* (Westring, 1861)

Nikolić and Polenec 1981, Polenec 1962a, 1968b, 1969a, 1970a

***Walckenaeria corniculans*** (O. P.-Cambridge, 1875)

Gorjan 2014

= *Prosopotheca corniculans* (Cambridge, 1875)

Nikolić and Polenec 1981, Polenec 1969a, 1978d, 1985, 1989

***Walckenaeria cucullata*** (C. L. Koch, 1836)

Gorjan 2014, Polenec 1957, 1959, 1960

= *Wideria cucullata* (C. L. Koch, 1836)

Bole et al. 1980, Nikolić and Polenec 1981, Polenec 1962a, 1964a, 1964b, 1965b, 1966b, 1969b, 1973d, 1974a, 1974b, 1975a, 1976a, 1978d, 1983, 1989

***Walckenaeria cuspidata*** Blackwall, 1833

= *Cornicularia cuspidata* (Blackwall, 1833)

Nikolić and Polenec 1981, Polenec 1970a, 1971b, 1979a, 1982a, 1989

***Walckenaeria furcillata*** (Menge, 1869)

Gorjan 2014, Gregorič and Kuntner 2009

= *Tigellinus furcillatus* (Menge, 1871)

Bole et al. 1980, Nikolić and Polenec 1981, Polenec 1965a, 1986, 1989

***Walckenaeria mitrata*** (Menge, 1868)

Gorjan 2014, Gregorič and Kuntner 2009, Polenec 1957, 1958, 1960, 1968b

= *Wideria mitrata* (Menge, 1868)

Bole et al. 1980, Nikolić and Polenec 1981, Polenec 1962a, 1964a, 1965a, 1968a, 1969a, 1970b, 1974a, 1975a, 1975b, 1977, 1983, 1985, 1989

***Walckenaeria monoceros*** (Wider, 1834)

= *Prosopotheca monoceros* (Wider, 1834)

Bole et al. 1980, Nikolić and Polenec 1981, Novak 2005b, Polenec 1962a, 1968b

***Walckenaeria obtusa*** Blackwall, 1836

Zupančič et al. 1984

= *Trachynella obtusa* (Blackwall, 1836)

Nikolić and Polenec 1981, Polenec 1962a, 1963e, 1965b, 1968a, 1975b, 1984, 1989

***Walckenaeria simplex*** Chyzer, 1894

= *Prosopotheca simplex* Miller, 1971

Bole et al. 1980

= *Wideria simplex* (Chyzer, 1894)

Nikolić and Polenec 1981, Polenec 1989

**Family LIOCRANIDAE**

***Agroeca brunnea*** (Blackwall, 1833)

Bole et al. 1980, Čandek et al. 2013, Gregorič and Kuntner 2009, Grimm and Bibel 1986, Nikolić and Polenec 1981, Polenec 1957, 1958, 1959, 1960, 1961a, 1962a, 1963e, 1964b, 1965a, 1965b, 1968a, 1973c, 1975b, 1976a, 1982a, 1989, Tarman 2003

***Agroeca cuprea*** Menge, 1873

Bole et al. 1980, Gorjan 2014, Gregorič and Kuntner 2009, Polenec 1957, 1958, 1963e, 1965b, 1978d

= *Agroeca pullata* Thorell, 1875

Grimm and Bibel 1986, Nikolić and Polenec 1981, Polenec 1962a, 1968b, 1969a, 1984, 1985, 1989

***Agroeca proxima*** (O. P.-Cambridge, 1871)

Bole et al. 1980

***Apostenus fuscus*** Westring, 1851

Grimm and Bibel 1986, Polenec 1960, 1964b, 1974a, 1975a, 1976a, 1978d, 1983, 1986, 1989

***Liocranum perarmatum*** Kulczyński, 1897

Nikolić and Polenec 1981, Polenec 1978d

***Liocranum rupicola*** (Walckenaer, 1830)

Čandek et al. 2013, Gregorič and Kuntner 2009, Grimm and Bibel 1986, Kuntner and Kostanjšek 2000, Nikolić and Polenec 1981, Novak 2005b, Polenec 1966b, 1989

***Sagana rutilans*** Thorell, 1875

= *Liocranum rutilans* (Thorell, 1875)

Gregorič and Kuntner 2009, Kuntner and Kostanjšek 2000, Polenec 1979a, 1989, 1992

***Scotina celans*** (Blackwall, 1841)

Bole et al. 1980, Grimm and Bibel 1986, Kuntner and Kostanjšek 2000, Nikolić and Polenec 1981, Novak 2005b, Polenec 1965b, 1976a, 1982a, 1989

***Scotina palliardii*** (L. Koch, 1881)

Bole et al. 1980, Polenec 1981, 1989

**Family LYCOSIDAE**

***Acantholycosa lignaria*** (Clerck, 1757)

Bole et al. 1980, Polenec 1992, Thaler and Buchar 1994

***Acantholycosa pedestris*** (Simon, 1876)

Nikolić and Polenec 1981, Polenec 1992, Tarman 2003

= *Acantholycosa strandi* (Kratochvíl, 1935)

Buchar and Polenec 1974, Polenec 1970a, 1971b

= *Lycosa longipes* Thorell, 1872

Polenec 1973a

= *Lycosa strandi* Kratochvíl, 1935

Kratochvíl 1935, Polenec 1973a, Růžička et al. 2005

***Alopecosa accentuata*** (Latreille, 1817)

Thaler and Buchar 1994

= *Alopecosa barbipes* (Sundevall, 1833)

Gorjan 2014, Nikolić and Polenec 1981, Polenec 1989

= *Tarentula barbipes* Sundevall, 1833

Polenec 1962a, 1968b, 1969a, 1970b, 1971c, 1975a

***Alopecosa aculeata*** (Clerck, 1757)

Gorjan 2014, Polenec 1978b, 1989, Zupančič et al. 1984

= *Tarentula aculeata* (Clerck, 1757)

Nikolić and Polenec 1981, Polenec 1957, 1958, 1963e, 1965a, 1966b, 1968a, 1969a, 1970a, 1970b, 1973c

***Alopecosa cuneata*** (Clerck, 1757)

Gorjan 2014, Gregorič and Kuntner 2009, Polenec 1975b, 1976b, 1978c, 1986, 1989

= *Tarentula cuneata* (Clerck, 1757)

Nikolić and Polenec 1981, Polenec 1969a, 1970a, 1970b, 1971c

***Alopecosa cursor*** (Hahn, 1831)

Polenec 1992

= *Alopecosa cursor cursor* 0

Bole et al. 1980

= *Tarentula cursor* (Hahn, 1831)

Nikolić and Polenec 1981

***Alopecosa fabrilis*** (Clerck, 1757)

Kuntner and Kostanjšek 2000

***Alopecosa inquilina*** (Clerck, 1757)

Bole et al. 1980, Polenec 1971b, 1989, 1992

= *Tarentula inquilina* (Clerck, 1757)

Nikolić and Polenec 1981, Polenec 1967a, 1970a, 1973b, 1974b

***Alopecosa mariae*** (Dahl, 1908)

Bole et al. 1980, Buchar and Thaler 2004, Gregorič and Kuntner 2009, Polenec 1992

= *Tarentula mariae* (Dahl, 1908)

Nikolić and Polenec 1981, Polenec 1968b, 1973e, 1975a

***Alopecosa pulverulenta*** (Clerck, 1757)

Bole et al. 1980, 1982, Čandek et al. 2013, Gorjan 2014, Gregorič and Kuntner 2009, Kostanjšek 2003, Kuntner 1997c, Polenec 1968b, 1975a, 1978d, 1980b, 1981, 1982a, 1989, Zupančič et al. 1984

= *Tarentula pulverulenta* (Clerck, 1757)

Nikolić and Polenec 1981, Polenec 1965b

***Alopecosa schmidti*** (Hahn, 1835)

Bole et al. 1980

= *Lycosa schmidti* Hahn, 1835

Polenec 1954, Reimoser 1919

***Alopecosa sulzeri*** (Pavesi, 1873)

Čandek et al. 2013, Gregorič and Kuntner 2009, Polenec 1978d

= *Tarentula sulzeri* (Pavesi, 1873)

Nikolić and Polenec 1981

***Alopecosa trabalis*** (Clerck, 1757)

Bole et al. 1980, 1982, Čandek et al. 2013, Gregorič and Kuntner 2009, Kuntner 1997d, Polenec 1975b, 1976b, 1977, 1978b, 1978c, 1978d, 1980a, 1980b, 1981, 1983, 1984, 1986, 1989, Zupančič et al. 1984

= *Tarentula trabalis* (Clerck, 1757)

Giltay 1932, Polenec 1967a, 1968a, 1969b, 1970a, 1970b, 1971c, 1973d, 1973e, 1974b

***Arctosa alpigena*** (Doleschall, 1852)

Tarman 2003

***Arctosa cinerea*** (Fabricius, 1777)

Kostanjšek 2010, Kuntner et al. 2007

***Arctosa figurata*** (Simon, 1876)

Gregorič and Kuntner 2009, Nikolić and Polenec 1981, Polenec 1968b, 1975a, 1992

***Arctosa fulvolineata*** (Lucas, 1846)

Čandek et al. 2013

***Arctosa leopardus*** (Sundevall, 1833)

Nikolić and Polenec 1981, Polenec 1976b, 1989, 1992

***Arctosa lutetiana*** (Simon, 1876)

Čandek et al. 2013, Gorjan 2014

= *Tricca lutetiana* Simon, 1889

Bole et al. 1982

***Arctosa maculata*** (Hahn, 1822)

Čandek et al. 2013, Kostanjšek 2004a, Polenec 1967a

***Arctosa perita*** (Latreille, 1799)

Gorjan 2014

***Arctosa personata*** (Koch, 1872)

Gorjan 2014, Kostanjšek 2002a

***Arctosa stigmosa*** (Thorell, 1875)

Polenec 1989, 1992

***Aulonia albimana*** (Walckenaer, 1805)

Bole et al. 1980, 1982, Čandek et al. 2013, Gorjan 2014, Gregorič and Kuntner 2009, Kuntner 1996, 1997d, Kuntner and Kostanjšek 2000, Nikolić and Polenec 1981, Polenec 1957, 1958, 1963e, 1964b, 1965a, 1965b, 1968b, 1969a, 1970b, 1971a, 1973c, 1973d, 1973e, 1974b, 1975a, 1975b, 1976a, 1976b, 1977, 1978d, 1982a, 1985, 1989

***Hogna radiata*** (Latreille, 1817)

Čandek et al. 2013, Gorjan 2014, Gregorič and Kuntner 2009, Kostanjšek 2004a, Kostanjšek and Kuntner 2002, Kuntner 1997d, 1999a, 1999b, 2001, Kuntner and Kostanjšek 2000, Nikolić and Polenec 1981

= *Lycosa radiata* (Latreille, 1817)

Polenec 1992, Thaler and Buchar 1994

= *Tarentula radiata* Latreille, 1872

Polenec 1968b

***Lycosa singoriensis*** (Laxmann, 1770)

Polenec 1992

***Pardosa agrestis*** (Westring, 1861)

Čandek et al. 2013, Kuntner and Kostanjšek 2000, Nikolić and Polenec 1981, Polenec 1989

= *Lycosa agrestis* Westring, 1861

Polenec 1970b

***Pardosa agricola*** (Thorell, 1856)

Kostanjšek 2010

***Pardosa alacris*** (C. L. Koch, 1833)

Töpfer-Hofmann et al. 2000

***Pardosa amentata*** (Clerck, 1757)

Bole et al. 1980, Čandek et al. 2013, Kostanjšek 2004a, Kuntner 1997c, Nikolić and Polenec 1981, Polenec 1976b, 1985, 1986, 1989, Tarman 2003, Tongiorgi 1966

= *Lycosa saccata* Linnaeus, 1758

Polenec 1970a, 1970b, 1971a, 1971c, 1973d, 1974a

***Pardosa bifasciata*** (C. L. Koch, 1834)

Čandek et al. 2013, Gorjan 2014, Gregorič and Kuntner 2009, Nikolić and Polenec 1981, Polenec 1968b, 1975a

= *Lycosa bifasciata* C. L. Koch, 1834

Polenec 1971a

***Pardosa blanda*** (C. L. Koch, 1833)

Bole et al. 1980, Nikolić and Polenec 1981, Polenec 1971b, 1980a, 1980b, 1981, 1989

= *Lycosa blanda* C. L. Koch, 1833

Polenec 1970a, 1973b

***Pardosa ferruginea*** (L. Koch, 1870)

Kuntner and Kostanjšek 2000, Nikolić and Polenec 1981, Polenec 1969a, 1971b, Zupančič et al. 1984

= *Lycosa ferruginea* L. Koch, 1870

Polenec 1970a

***Pardosa hortensis*** (Thorell, 1872)

Čandek et al. 2013, Gorjan 2014, Kostanjšek 2003, Kuntner 1997d, Nikolić and Polenec 1981, Polenec 1989, Tongiorgi 1966

= *Lycosa hortensis* Thorell, 1872

Polenec 1970b, 1971a

***Pardosa lugubris*** (Walckenaer, 1802)

Bole et al. 1980, 1982, Budja 2008, Čandek et al. 2013, Gorjan 2014, Gregorič and Kuntner 2009, Kostanjšek 2003, 2004a, Kuntner 1996, 1997c, 1997d, 1999b, Kuntner and Baxter 1997, Kuntner and Kostanjšek 2000, Nikolić and Polenec 1981, Novak 2005b, Polak 1997, Polenec 1975b, 1976a, 1977, 1978d, 1979a, 1983, 1984, 1985, 1986, 1989, Tarman 2003, Tongiorgi 1966, Zupančič et al. 1984

= *Lycosa chelata* O.F. Müller

Bole et al. 1980, Novak 1981, Polenec 1957, 1958, 1959, 1960, 1963d, 1963e, 1964a, 1964b, 1965a, 1965b, 1969a, 1969b, 1970b, 1971a, 1973c, 1973d, 1973e, 1974a, 1975a

***Pardosa monticola*** (Clerck, 1757)

Kuntner 1997d, Nikolić and Polenec 1981, Polenec 1969a, 1980b, 1989

= *Lycosa monticola* Clerck

Polenec 1963e, 1970a, 1971a, 1973d, 1973e

***Pardosa morosa*** (L. Koch, 1870)

Nikolić and Polenec 1981, Polenec 1992

***Pardosa nigra*** (C. L. Koch, 1834)

Nikolić and Polenec 1981, Polenec 1971b, 1973a, Tarman 2003

= *Lycosa ludovici* Dahl, 1908

Kratochvíl 1935, Polenec 1970a

= *Lycosa nigra* C. L. Koch, 1834

Doleschall 1852

***Pardosa palustris*** (Linnaeus, 1758)

Bole et al. 1980, Čandek et al. 2013, Kostanjšek 2004a, Nikolić and Polenec 1981, Polenec 1976b, 1980a, 1989, 1992, Zupančič et al. 1984

= *Lycosa tarsalis* Thorell, 1856

Polenec 1967a, 1970a, 1970b

***Pardosa prativaga*** (L. Koch, 1870)

Kostanjšek 2010, 2014

***Pardosa proxima*** (C. L. Koch, 1847)

Čandek et al. 2013, Kostanjšek 2010

***Pardosa pseudostrigillata*** Tongiorgi, 1966

Buchar and Thaler 2002

***Pardosa pullata*** (Clerck, 1757)

Nikolić and Polenec 1981, Polenec 1971b, 1976a, 1989, Tarman 2003

= *Lycosa pullata* Clerck

Polenec 1970a, 1970b, 1971a, 1973b, 1973d

***Pardosa riparia*** (C. L. Koch, 1833)

Bole et al. 1980, 1982, Čandek et al. 2013, Gregorič and Kuntner 2009, Nikolić and Polenec 1981, Polenec 1971b, 1976a, 1978c, 1980a, 1981, 1989, Zupančič et al. 1984

= *Alopecosa riparia* (C. L. Koch, 1833)

Polenec 1975b

= *Lycosa cursoria* C. L. Koch, 1847

Polenec 1967a, 1969a, 1969b, 1970a, 1971c, 1973b, 1973e, 1974b

= *Pardosa cursoria* (C. L. Koch, 1847)

Bole et al. 1980, Polenec 1969a

***Pardosa saltuaria*** (L. Koch, 1870)

Bole et al. 1980, Nikolić and Polenec 1981, Polenec 1968a, 1971b, 1978b, 1980b, 1981, 1982a, 1989, Zupančič et al. 1984

= *Lycosa saltuaria* L. Koch, 1870

Polenec 1970a, 1973b, 1974b

***Pardosa saturatior*** Simon, 1937

Buchar and Thaler 2002, Kuntner 1999b, 2001, 2002, ULRS 2002

***Pardosa vittata*** (Keyserling, 1863)

Nikolić and Polenec 1981, Polenec 1974a, 1992

***Pardosa wagleri*** (Hahn, 1822)

Polenec 1992

***Pirata piraticus*** (Clerck, 1757)

Kostanjšek 2002b, 2011, 2014, Kuntner 1999b, 2001, 2002, Tarman 2003, Tovornik 1983, 1990, ULRS 2002

***Pirata tenuitarsis*** Simon, 1876

Kuntner 1997c, 2001, 2002, ULRS 2002

***Pirata uliginosus*** (Thorell, 1856)

Kostanjšek 2013, 2014, Kuntner 1997c, 1999b, 2001, 2002, ULRS 2002

***Piratula hygrophyla*** (Thorell, 1872)

Čandek et al. 2013

= *Pirata hydrophilus* Thorell, 1872

Polenec 1975b, 1986, 1989

= *Pirata hygrophila* (Thorell, 1872)

Kuntner 1997c, Nikolić and Polenec 1981

***Piratula knorri*** (Scopoli, 1763)

Čandek et al. 2013

= *Pirata knorri* (Scopoli, 1763)

Buchar and Polenec 1974, Kostanjšek 2001, 2002a, 2004a, Nikolić and Polenec 1981, Polenec 1954, 1989, 1992, Tarman 2003

***Piratula latitans*** (Blackwall, 1841)

Nikolić and Polenec 1981

= *Pirata latitans* (Blackwall, 1841)

Kuntner 1997d, Polenec 1976b, 1986, 1989

***Trochosa hispanica*** Simon, 1870

Bole et al. 1980

= *Trochosa manicata* Thorell, 1875

Bole et al. 1980

***Trochosa robusta*** (Simon, 1876)

Nikolić and Polenec 1981, Polenec 1978d, 1981

***Trochosa ruricola*** (De Geer, 1778)

Gregorič and Kuntner 2009, Polenec 1969a, 1976b, 1978d, 1989

= *Trochosa lapidicola* Hahn, 1829

Polenec 1965b

***Trochosa spinipalpis*** (F. O. P.- Cambridge, 1895)

Čandek et al. 2013, Nikolić and Polenec 1981, Polenec 1957, 1958, 1973c, 1989, 1992

***Trochosa terricola*** Thorell, 1856

Bole et al. 1980, 1982, Budja 2008, Gregorič and Kuntner 2009, Kostanjšek 2003, 2004a, Kuntner 1997d, 1999b, Kuntner and Kostanjšek 2000, Nikolić and Polenec 1981, Novak 2005b, Polenec 1957, 1958, 1959, 1960, 1961d, 1962a, 1963e, 1964b, 1965a, 1965b, 1966b, 1967a, 1967b, 1968a, 1968b, 1969a, 1970a, 1971a, 1971c, 1973c, 1973d, 1973e, 1974b, 1975b, 1976a, 1976b, 1977, 1978b, 1978c, 1978d, 1979a, 1980b, 1981, 1982a, 1983, 1984, 1985, 1986, 1987, 1989, Tarman 2003, Zupančič et al. 1984

***Xerolycosa nemoralis*** (Westring, 1861)

Buchar and Dolansky 2011, Budja 2008, Kuntner 1997d, Nikolić and Polenec 1981, Polenec 1967a, 1970a, 1971a, 1971b, 1973b, 1974a, 1981, 1983, 1986, 1989

**Family MIMETIDAE**

***Ero aphana*** (Walckenaer, 1802)

Kuntner 1997d, 2002, Le Peru 2011

***Ero furcata*** (Villers, 1789)

Bole et al. 1980, Gorjan 2014, Gregorič and Kuntner 2009, Kuntner 1997d, Le Peru 2011, Nikolić and Polenec 1981, Polenec 1957, 1958, 1963e, 1971a, 1974a, 1975a, 1982a, 1983, 1989

***Ero tuberculata*** (De Geer, 1778)

Kostanjšek 2005, Kuntner 1999b, Le Peru 2011, Nikolić and Polenec 1981, Polenec 1978d

**Family MITURGIDAE**

***Zora armillata*** Simon, 1878

Kostanjšek 2010, 2014

***Zora nemoralis*** (Blackwall, 1861)

Bole et al. 1980, Gregorič and Kuntner 2009, Kuntner 1997c, Nikolić and Polenec 1981, Polenec 1964b, 1977, 1978d, 1984, 1985, 1989

***Zora spinimana*** (Sundevall, 1833)

Bole et al. 1980, 1982, Čandek et al. 2013, Gorjan 2014, Gregorič and Kuntner 2009, Kuntner 1997d, 1999b, Nikolić and Polenec 1981, Polenec 1957, 1958, 1963e, 1964b, 1965b, 1969a, 1971a, 1981, 1985, 1989

**Family MYSMENIDAE**

***Mysmenella jobi*** (Kraus, 1967)

Le Peru 2011

= *Mysmena jobi* Kraus, 1967

Nikolić and Polenec 1981, Polenec 1992

Trogloneta granulum* Simon, 1922

1♂ - chasm Unška Koliševka, central Slovenia; 45°18,81'N, 14°15,81'E, 600 m a. s. l.; 1.9.2000; leg. & det. Řezáč M., Kuntner M., Polak S.

**Family NEMESIIDAE**

***Brachythele media*** Kulczyński, 1897

Bole et al. 1980, 1982, Le Peru 2011, Nikolić and Polenec 1981, Polenec 1978a, 1978d, 1979b, Tarman 2003

***Nemesia caementaria*** (Latreille, 1799)

Reimoser 1919

= *Nemesia caementaria germanica* Ausserer, 1871

Polenec 1978a, 1992, Tarman 2003

***Nemesia pannonica*** Herman, 1879

Čandek et al. 2013, Deceae 2010, Gorjan 2014, Gregorič and Kuntner 2009, Kostanjšek 2003, Le Peru 2011, Polenec 1966a, 1968b, 1978a, 1978d, 1979b, Tarman 2003

= *Nemesia pannonica pannonica* Herman, 1879

Fuhn and Polenec 1967, Nikolić and Polenec 1981, Polenec 1975a, 1978a

**Family NESTICIDAE**

***Nesticus cellulanus*** (Clerck, 1757)

Brignoli 1971b, 1980, Caporiacco 1938, Čandek et al. 2013, Drensky 1936, Gasparo and Thaler 2000, Kostanjšek 2004a, Kratochvíl 1933, 1936, Kuntner 1997d, 1999a, 1999b, Le Peru 2011, Novak 1981, 2005b, Novak and Kuštor 1982, Novak and Sivec 1976, Novak et al. 2004, Polak 1994, Polak et al. 2012, Polenec 1973b, 1989, 1992, Staut et al. 2008, Tarman 2003, Thaler 1981a

***Nesticus eremita*** Simon, 1879

Brignoli 1971b, 1980, Kostanjšek 2003, Le Peru 2011, Polak et al. 2012

= *Nesticus speluncarum eremita* E. Simon, 1879

Kratochvíl 1934

***Nesticus speluncarum*** Pavesi, 1873

Le Peru 2011, Nikolić 1963, Nikolić and Polenec 1981

***Typhlonesticus idriacus*** (Roewer, 1931)

= *Nesticus idriacus* Roewer, 1931

Deeleman-Reinhold 1975, 1978, 1980, 1981, Komposch and Steinberger 1999, Kostanjšek 2001, 2010, Kranjc and Novak 1978, Kratochvíl 1933, Le Peru 2011, Nikolić 1957, 1961, Nikolić and Polenec 1981, Novak 2005b, Polenec 1992, Roewer 1931, Thaler 1976, 1981a, ULRS 2002, 2004

**Family OECOBIIDAE**

***Oecobius maculatus*** Simon, 1870

Kostanjšek and Gorjan 2013

***Uroctea durandi*** (Latreille, 1809)

Čandek et al. 2013, Kostanjšek 2003, 2005, Kuntner 1997a, Le Peru 2011, Nikolić and Polenec 1981, Polenec 1992, Tarman 2003

**Family OXYOPIDAE**

***Oxyopes heterophthalmus*** (Latreille, 1804)

Kostanjšek 2000, 2003, Kuntner 1997a, 2001, 2002, ULRS 2002

***Oxyopes lineatus*** Latreille, 1806

Gregorič and Kuntner 2009, Kostanjšek and Kuntner 2002, Kuntner 1997d, 2001, 2002, Kuntner and Kostanjšek 2000

***Oxyopes ramosus*** (Martini & Goeze, 1778)

Brignoli 1976, Kostanjšek 2000, Nikolić and Polenec 1981, Polenec 1957, 1958

**Family PHILODROMIDAE**

***Philodromus albidus*** Kulczyński, 1911

Budja 2008, Čandek et al. 2013, Kuntner 1997c, 1997d, 1999b, 2002, Kuntner and Kostanjšek 2000

***Philodromus aureolus*** (Clerck, 1757)

Čandek et al. 2013, Kuntner 1997d, Kuntner and Kostanjšek 2000, Polenec 1957, 1958, 1973c, 1989

***Philodromus cespitum*** (Walckenaer, 1802)

Čandek et al. 2013, Kostanjšek 2004a, Kuntner 2002, Kuntner and Kostanjšek 2000

= *Philodromus aureolus caespiticolis* (Walckenaer, 1837)

Nikolić and Polenec 1981, Polenec 1969a

***Philodromus collinus*** C. L. Koch, 1835

Kuntner 1997c, Polenec 1967b, 1984, 1985, 1989, Šilhavy 1944

***Philodromus dispar*** Walckenaer, 1826

Budja 2008, Čandek et al. 2013, Kuntner 1999b, Kuntner and Kostanjšek 2000, Nikolić and Polenec 1981, Novak 1981, 2005b, Polenec 1957, 1958

***Philodromus fuscolimbatus*** Lucas, 1846

= *Philodromus buxi constellatus* (Simon, 1875)

Nikolić and Polenec 1981

***Philodromus fuscomarginatus*** (De Geer, 1778)

Polenec 1970a

***Philodromus longipalpis*** Simon, 1870

Budja 2008, Kostanjšek 2010, 2011

= *Philodromus aureolus pallens* Klulczinsky

Šilhavy 1944

***Philodromus margaritatus*** (Clerck, 1757)

Kostanjšek 2013, Šilhavy 1944, Tarman 2003

***Philodromus poecilus*** (Thorell, 1872)

Kostanjšek 2003

***Philodromus praedatus*** O. P.-Cambridge, 1871

Čandek et al. 2013, Kostanjšek and Celestina 2008

***Philodromus pulchellus*** Lucas, 1846

Čandek et al. 2013

***Philodromus rufus*** Walckenaer, 1826

Kuntner 1997d, 2002

***Thanatus arenarius*** L. Koch, 1872

Gregorič and Kuntner 2009

Thanatus atratus* Simon, 1875

20♂ - meadow on W slope of Sv. Katarin hill, N of Nova Gorica; 45°57,96'N, 13°39,51'E, 190 m a. s. l.; 14.6-5.7.2008; leg. Gorjan A. & det. Gorjan A., Kostanjšek R.

***Thanatus formicinus*** (Clerck, 1757)

Bole et al. 1980, Čandek et al. 2013, Gregorič and Kuntner 2009, Polenec 1980b, 1985, 1989

***Tibellus macellus*** Simon, 1875

Čandek et al. 2013

***Tibellus oblongus*** (Walckenaer, 1802)

Čandek et al. 2013, Gregorič and Kuntner 2009, Kuntner and Kostanjšek 2000, Nikolić and Polenec 1981, Polenec 1969a

**Family PHOLCIDAE**

***Holocnemus pluchei*** (Scopoli, 1763)

Kostanjšek and Kuntner 2002, Kuntner 2001, 2002, Kuntner and Kostanjšek 2000, Le Peru 2011, Polenec 1954, 1992, ULRS 2002

= *Aranea pluchii* Scopoli, 1763

Scopoli 1763

***Pholcus opilionoides*** (Schrank, 1781)

Bole et al. 1980, Čandek et al. 2013, Kostanjšek 2004a, Kuntner 1997d, Kuntner and Kostanjšek 2000, Nikolić and Polenec 1981, Polenec 1989, Tarman 2003

***Pholcus phalangioides*** (Fuesslin, 1775)

Brignoli 1971a, Čandek et al. 2013, Kostanjšek 2004a, 2012, Kratochvíl 1934, Kuntner 1996, 1997d, 1999b, Kuntner and Kostanjšek 2000, Nikolić and Polenec 1981, Novak 2005b, Novak et al. 2004, Polenec 1962b, 1966b, 1989, Stražar 2001, Tarman 2003

***Psilochorus simoni*** (Berland, 1911)

Čandek et al. 2013, Kostanjšek 2012, Kostanjšek and Ramšak 2005

***Spermophora senoculata*** (Dugès, 1836)

Kuntner 1997d, 2001, 2002, Le Peru 2011, Nentwig and Kobelt 2010, ULRS 2002

**Family PHRUROLITHIDAE**

***Phrurolithus festivus*** (C. L. Koch, 1835)

Bole et al. 1980, Čandek et al. 2013, Gorjan 2014, Gregorič and Kuntner 2009, Grimm and Bibel 1986, Kuntner 1999b, Nikolić and Polenec 1981, Polenec 1957, 1958, 1963e, 1965a, 1965b, 1968b, 1973c, 1975a, 1989

***Phrurolithus minimus*** C. L. Koch, 1839

Čandek et al. 2013, Gorjan 2014, Grimm and Bibel 1986, Nikolić and Polenec 1981, Polenec 1957, 1958, 1965a, 1973c, 1975b, 1984, 1989

**Family PISAURIDAE**

***Dolomedes fimbriatus*** (Clerck, 1757)

Brignoli 1976, Kostanjšek 2003, Kuntner 1997c, Nikolić and Polenec 1981, Polenec 1957, 1958, 1963d, 1969b, 1971c, 1973c, 1974a, 1986, 1989, 1992, Tarman 2003, Tovornik 1983, 1990, Vogrin 2002

***Dolomedes plantarius*** (Clerck, 1757)

Kostanjšek 2014, Nikolić and Polenec 1981, Polenec 1992, ULRS 1993, 2002

***Pisaura mirabilis*** (Clerck, 1757)

Brignoli 1976, Čandek et al. 2013, Gregorič and Kuntner 2009, Kostanjšek 2003, 2004a, Kuntner 1996, 1997c, 1997d, 1999b, Kuntner and Kostanjšek 2000, Neuhauser et al. 1995, Nikolić and Polenec 1981, Polenec 1978d, 1984, 1985, 1989, Tarman 2003

= *Pisaura listeri* Scopoli, 1763

Bole et al. 1980, Polenec 1952, 1954, 1957, 1958, 1963c, 1963e, 1964a, 1965a, 1965b, 1967b, 1969a, 1969b, 1970b, 1971a, 1975b

**Family SALTICIDAE**

***Aelurillus v-insignitus*** (Clerck, 1757)

Gorjan 2014, Kostanjšek and Fišer 2005

***Asianellus festivus*** (C. L. Koch, 1834)

Fišer and Kostanjšek 2001, Gorjan 2014

***Ballus chalybeius*** (Walckenaer, 1802)

Gorjan 2014, Kuntner and Kostanjšek 2000, Nikolić and Polenec 1981

***Carrhotus xanthogramma*** (Latreille, 1819)

Kuntner 1997b, 1997d, 2002, Kuntner and Kostanjšek 2000, Tarman 2003

***Chalcoscirtus infimus*** (Simon, 1868)

Fišer and Kostanjšek 2001

***Dendryphantes hastatus*** (Clerk, 1757)

Kuntner 1997b, Nikolić and Polenec 1981

= *Dendryphantes pini* Hahn, 1831

Polenec 1957, 1958

***Dendryphantes rudis*** (Sundevall, 1833)

Kuntner 1997b, 1997c, 1999b, 2002

***Euophrys frontalis*** (Walckenaer, 1802)

Bole et al. 1980, Gorjan 2014, Kuntner 1997d, Polenec 1963e, 1968b, 1975a, 1978c, 1978d, 1981, 1986

= *Euophrys maculata* (Wider, 1834)

Nikolić and Polenec 1981, Polenec 1957, 1958, 1985, 1989

***Euophrys herbigrada*** (Simon, 1871)

Fišer and Kostanjšek 2001, Gorjan 2014, Gregorič and Kuntner 2009

***Evarcha arcuata*** (Clerck, 1757)

Budja 2008, Čandek et al. 2013, Gregorič and Kuntner 2009, Knapič 2012, Kostanjšek 2003, 2004a, Kuntner 1996, 1997c, 1997d, 1999b, Kuntner and Kostanjšek 2000, Nikolić and Polenec 1981, Polenec 1989

= *Aranea marcgravii* Scopoli, 1763

Scopoli 1763

= *Attus albociliatus* Simon, 1868

Simon 1868

= *Evarcha marcgravi* Scopoli

Polenec 1954, 1957, 1958, 1963e, 1969b, 1970b, 1971a, 1973c, 1975b, 1976b

= *Evarcha meregravi* Scopoli

Polenec 1965a

***Evarcha falcata*** (Clerck, 1757)

Bole et al. 1980, Budja 2008, Čandek et al. 2013, Gregorič and Kuntner 2009, Knapič 2012, Kostanjšek 2003, 2004a, Kuntner 1996, 1999b, Kuntner and Kostanjšek 2000, Polak 1997, Polenec 1977, 1978d, 1980a

= *Aranea blancardi* Scopoli, 1763

Scopoli 1763

= *Attus falcatus* Simon, 1868

Simon 1868

= *Evarcha blancardi* Scopoli, 1763

Bole et al. 1980, Polenec 1954, 1957, 1958, 1963d, 1964b, 1965a, 1967b, 1970a, 1973c, 1973e, 1974b, 1980a

= *Evarcha flammata* (Clerck, 1757)

Kuntner 1997c, 1997d, Nikolić and Polenec 1981, Polenec 1977, 1989

***Evarcha jucunda*** (Lucas, 1846)

Čandek et al. 2013, Gorjan 2014, Knapič 2012, Kuntner 1997b, 2001, 2002, Polenec 1992, ULRS 2002

***Evarcha laetabunda*** (C. L. Koch, 1846)

Čandek et al. 2013, 2015, Gorjan 2014, Gregorič and Kuntner 2009, Knapič 2012, Kuntner 1997d, Nikolić and Polenec 1981, Polenec 1971a, 1978d

***Evarcha michailovi*** Logunov, 1992

Čandek et al. 2013, 2015, Fišer and Kostanjšek 2001, Knapič 2012

***Hasarius adansoni*** (Audouin, 1826)

Čandek et al. 2013, Kostanjšek 2002b

***Heliophanus aeneus*** (Hahn, 1832)

Čandek et al. 2013, Fišer and Kostanjšek 2001, Kostanjšek 2003

***Heliophanus auratus*** C. L. Koch, 1835

Gregorič and Kuntner 2009, Kuntner 1997d, Kuntner and Kostanjšek 2000, Nikolić and Polenec 1981, Polenec 1971a

***Heliophanus cupreus*** (Walckenaer, 1802)

Čandek et al. 2013, Gorjan 2014, Gregorič and Kuntner 2009, Kostanjšek 2003, 2004a, Kuntner 1997b, 1997d, 1999b, 2002, Kuntner and Kostanjšek 2000

***Heliophanus dubius*** C. L. Koch, 1835

Fišer and Kostanjšek 2001, Kostanjšek 2001, Tarman 2003

***Heliophanus flavipes*** (Hahn, 1832)

Čandek et al. 2013, Kostanjšek 2001, 2004a, Kuntner 1999b

= *Aranea ritteri* Scopoli, 1763

Scopoli 1763

= *Heliophanus ritteri* (Scopoli, 1763)

Nikolić and Polenec 1981, Polenec 1954, 1971a, 1989

***Heliophanus kochii*** Simon, 1868

Čandek et al. 2013, Fišer and Kostanjšek 2001, Gorjan 2014

***Heliophanus lineiventris*** Simon, 1868

= *Heliophanus pouzdranensis* Miller, 1958

Polenec 1992

***Heliophanus melinus*** L. Koch, 1867

Fišer and Kostanjšek 2001

***Heliophanus patagiatus*** Thorell, 1875

Fišer and Kostanjšek 2001

***Heliophanus tribulosus*** Simon, 1868

Fišer and Kostanjšek 2001, Gorjan 2014

***Icius hamatus*** (C. L. Koch, 1846)

Fišer and Kostanjšek 2001

***Icius subinermis*** Simon, 1937

Čandek et al. 2013, Kostanjšek and Fišer 2005

***Leptorchestes berolinensis*** (C. L. Koch, 1846)

Čandek et al. 2013, Kostanjšek 2003, Kuntner 1997a, 2002

= *Leptorchestes cinctus* Thorell, 1869

Polenec 1982a, 1989

***Macaroeris nidicolens*** (Walckenaer, 1802)

Čandek et al. 2013, Kuntner 2002

= *Eris nidicolens* (Walckenaer, 1802)

Kuntner 1997b, 1997d, Kuntner and Kostanjšek 2000

***Marpissa muscosa*** (Clerck, 1757)

Budja 2008, Čandek et al. 2013, Gregorič and Kuntner 2009, Kuntner 1996, 1999b, Kuntner and Baxter 1997, Nikolić and Polenec 1981, Polenec 1988, 1989

= *Aranea rumpfii* Scopoli, 1763

Scopoli 1763

= *Marpissa rumpfi* Scopoli

Polenec 1954

= *Marpissus muscosus* Simon, 1868

Simon 1868

***Marpissa nivoyi*** (Lucas, 1846)

Čandek et al. 2013, Franc 2004, Gregorič and Kuntner 2009, Kuntner 1997b, 2002, Polenec 1992

***Marpissa pomatia*** (Walckenaer, 1802)

Fišer and Kostanjšek 2001

***Mendoza canestrinii*** (Ninni, 1868)

Fišer and Kostanjšek 2001

***Menemerus semilimbatus*** (Hahn, 1829)

Kuntner 1997b, 2001, 2002, ULRS 2002

***Myrmarachne formicaria*** (De Geer, 1778)

Caporiacco 1949, Čandek et al. 2013, Gogala 1985, Gorjan 2014, Gregorič and Kuntner 2009

= *Aranea joblotii* Scopoli, 1763

Scopoli 1763

= *Myrmarachne joblotii* (Scopoli, 1763)

Nikolić and Polenec 1981, Polenec 1954, Tarman 2003

***Neon levis*** (Simon, 1871)

Bole et al. 1982, Gorjan 2014

***Neon rayi*** (Simon, 1875)

Fišer and Kostanjšek 2001

***Neon reticulatus*** (Blackwall, 1853)

Budja 2008, Čandek et al. 2013, Gorjan 2014, Kostanjšek 2004a, Nikolić and Polenec 1981, Polenec 1974a, 1975a

***Pellenes nigrociliatus*** (Simon, 1875)

Fišer and Kostanjšek 2001, Gorjan 2014

***Pellenes seriatus*** (Thorell, 1875)

Čandek et al. 2013, 2015, Fišer and Kostanjšek 2001, Gregorič and Kuntner 2009

***Pellenes tripunctatus*** (Walckenaer, 1802)

Bole et al. 1982, Franc 2004, Kostanjšek 2003, Polenec 1992

***Philaeus chrysops*** (Poda, 1761)

Čandek et al. 2013, Franc 2004, Gorjan 2014, Kostanjšek 2003, Kuntner 1997d, Kuntner and Kostanjšek 2000, Nikolić and Polenec 1981, Polenec 1966a, 1969a, 1992

= *Aranea catesbaei* Scopoli, 1763

Scopoli 1763

= *Aranea sloanii* Scopoli, 1763

Scopoli 1763

***Phlegra bresnieri*** (Lucas, 1846)

Kostanjšek 2003, Kostanjšek and Fišer 2005

***Phlegra cinereofasciata*** (Simon, 1868)

Kostanjšek and Fišer 2005

= *Phlegra fuscipes* Kulczyński, 1891

Bole et al. 1982

***Phlegra fasciata*** (Hahn, 1826)

Gorjan 2014, Gregorič and Kuntner 2009, Nikolić and Polenec 1981, Polenec 1968b, 1971a, 1975a

***Pseudeuophrys erratica*** (Walckenaer, 1826)

Fišer and Kostanjšek 2001

***Pseudeuophrys lanigera*** (Simon, 1871)

Čandek et al. 2013, Gregorič and Kuntner 2009, Kuntner 2001, 2002, ULRS 2002

= *Euophrys lanigera* (Simon, 1871)

Kuntner and Kostanjšek 2000

***Pseudeuophrys obsoleta*** (Simon, 1868)

Gorjan 2014, Gregorič and Kuntner 2009

= *Euophrys pictilis* (Simon, 1871)

Polenec 1992

***Pseudeuophrys vafra*** (Blackwall, 1867)

Kostanjšek and Fišer 2005

***Pseudicius encarpatus*** (Walckenaer, 1802)

Fišer and Kostanjšek 2001

***Saitis barbipes*** (Simon, 1868)

Bole et al. 1980, Čandek et al. 2013, Gorjan 2014, Kostanjšek 2003, Nikolić and Polenec 1981, Polenec 1978d, 1979b

***Salticus scenicus*** (Clerck, 1757)

Čandek et al. 2013, Nikolić and Polenec 1981, Polenec 1967b, Tarman 2003

***Salticus unciger*** (Simon, 1868)

Kostanjšek and Fišer 2005

***Salticus zebraneus*** (C. L. Koch, 1837)

= *Aranea olearii* Scopoli, 1763

Scopoli 1763

= *Salticus oleari* (Scopoli, 1763)

Nikolić and Polenec 1981, Polenec 1954, 1958, 1973c, 1989

***Sibianor aurocinctus*** (Ohlert, 1865)

Gorjan 2014

= *Bianor aenescens* Simon, 1868

Bole et al. 1980, Polenec 1975b, 1978d

= *Bianor aurocinctus* (Ohlert, 1865)

Kuntner 1997d, Nikolić and Polenec 1981, Polenec 1989

***Sitticus atricapillus*** (Simon, 1882)

= *Sitticus zimmermanni* (Simon, 1877)

Bole et al. 1982, Polenec 1982a, 1989, 1992

***Sitticus floricola*** (C. L. Koch, 1837)

Kuntner 1999b, 2002

= *Sitticus littoralis* (Hahn, 1832)

Polenec 1992, Tovornik 1990

***Sitticus inexpectus*** Logunov & Kronestedt, 1997

Kostanjšek and Fišer 2005

***Sitticus pubescens*** (Fabricius, 1775)

Franc 2004, Kostanjšek 2003, Polenec 1989, 1992

= *Sitticus truncorum* (Linnaeus, 1758)

Polenec 1988

***Sitticus saxicola*** (C. L. Koch, 1846)

Kuntner and Kostanjšek 2000, Polenec 1989

***Sitticus terebratus*** (Clerck, 1757)

Kostanjšek and Fišer 2005

***Synageles hilarulus*** (C. L. Koch, 1846)

Fišer and Kostanjšek 2001

***Synageles venator*** (Lucas, 1836)

Fišer and Kostanjšek 2001, Kostanjšek 2003

***Talavera aequipes*** (O. P.-Cambridge, 1871)

Gorjan 2014

= *Euophrys aequipes* (Cambridge, 1871)

Kostanjšek 2003, Nikolić and Polenec 1981, Polenec 1957, 1958, 1973c, 1989

***Talavera petrensis*** (C. L. Koch, 1837)

Gregorič and Kuntner 2009

= *Euophrys petrensis* C. L. Koch, 1837

Nikolić and Polenec 1981, Polenec 1957, 1958, 1959, 1965a, 1968b, 1973c, 1973e, 1989

***Talavera thorelli*** (Kulczyński, 1891)

Fišer and Kostanjšek 2001

**Family SCYTODIDAE**

***Scytodes thoracica*** (Latreille, 1802)

Čandek et al. 2013, Gorjan 2014, Gregorič and Kuntner 2009, Kostanjšek 2005, Kuntner 1997a, Le Peru 2011, Nikolić and Polenec 1981, Polenec 1988, 1989, 1992, Tarman 2003

**Family SEGESTRIIDAE**

***Segestria bavarica*** C. L. Koch, 1843

Gregorič and Kuntner 2009, Kuntner 1997d, 1999a, 1999b, Kuntner and Kostanjšek 2000, Le Peru 2011, Nikolić and Polenec 1981, Polenec 1989, 1992, Tarman 2003, Zupančič et al. 1984

***Segestria cavernicola*** Kulczyński, 1915

Le Peru 2011

***Segestria croatica*** Doleschall, 1852

Bole et al. 1982, Kulczyński 1915

***Segestria florentina*** (Rossi, 1790)

Kostanjšek and Kuntner 2002, Kuntner 1997a, 2002, Kuntner and Kostanjšek 2000, Le Peru 2011, Tarman 2003

***Segestria senoculata*** (Linnaeus, 1758)

Budja 2008, Čandek et al. 2013, Kostanjšek 2004a, Kuntner 1996, 1997d, Kuntner and Baxter 1997, Le Peru 2011, Nikolić and Polenec 1981, Novak 1981, 2005b, Polenec 1960, 1963e, 1964b, 1976b, 1978c, 1979a, 1982b, 1985, 1986, 1987, 1989

**Family SPARASSIDAE**

***Micrommata virescens*** (Clerck, 1757)

Čandek et al. 2013, Gregorič and Kuntner 2009, Kostanjšek 2003, 2004a, Kuntner 1996, 1997c, 1997d, 1999b, Kuntner and Kostanjšek 2000, Polenec 1964a, 1978d

= *Micrommata roseum* (Clerck, 1757)

Nikolić and Polenec 1981, Polenec 1985, 1986, 1989, Tarman 2003

= *Micrommata viridissima* De Geer

Bole et al. 1980, Polenec 1957, 1958, 1965a, 1969b, 1971a, 1971c, 1973c

***Micrommata virescens ornata*** (Walckenaer, 1802)

= *Micrommata roseum ornatum* (Walckenaer, 1802)

Nikolić and Polenec 1981

= Micrommata viridissima var. ornata Walckenaer

Polenec 1969a, 1974a

**Family TETRAGNATHIDAE**

***Meta menardi*** (Latreille, 1804)

Batič et al. 1980, Brignoli 1971b, 1980, Caporiacco 1938, Čandek et al. 2013, Deeleman-Reinhold 1978, Ferk and Lipar 2011, Kiauta and Leben 1960, Kostanjšek 2007, Kratochvíl 1934, 1936, Kuntner et al. 2008, Nikolić 1963, Nikolić and Polenec 1981, Novak 1971, 1981, 2005a, 2005b, Novak and Kuštor 1982, Novak and Sivec 1976, Novak et al. 2004, 2010, Polak 1994, 1997, Polak et al. 2012, Polenec 1962b, 1973b, Preisinger 2010, Puc et al. 1988, Rebeušek et al. 2008, Roewer 1931, Stražar 2001, Tarman 2003, Tkavc 2008

= *Epeira fusca* Walckenaer

Joseph 1881, Pokorny 1853, Schiner 1854

***Metellina mengei*** (Blackwall, 1870)

Budja 2008, Kostanjšek 2004a, Kuntner 1997c, 1997d, Kuntner and Kostanjšek 2000, Polak 1997

= *Meta mengei* (Blackwall, 1870)

Polenec 1976a

= Meta retuculata var. mengei Blackwall, 1869

Polenec 1958, 1963d, 1967b, 1969a, 1969b

= *Meta segmentata mengei* (Blackwall, 1869)

Nikolić and Polenec 1981, Polenec 1960, 1989

***Metellina merianae*** (Scopoli, 1763)

Budja 2008, Čandek et al. 2013, 2015, Kostanjšek 2003, 2004a, Kuntner 1997c, 1997d, 1999b, Kuntner and Kostanjšek 2000, Novak 2005a, Polak 1994, 1997, Rebeušek et al. 2008, Stražar 2001, Tarman 2003

= *Aranea merianae* Scopoli, 1763

Scopoli 1763

= *Meta merianae* (Scopoli, 1863)

Batič et al. 1980, Brignoli 1971b, 1980, Caporiacco 1938, Čandek et al. 2013, Fage 1931, Kratochvíl 1934, Nikolić 1963, Nikolić and Polenec 1981, Novak 1981, 2005b, Novak and Kuštor 1982, Novak and Sivec 1976, Novak et al. 2004, 2010, Polenec 1954, 1967b, 1971c, 1989, Puc et al. 1988, Sket 1999, Spazzapan-Brelih 1964

***Metellina segmentata*** (Clerck, 1757)

Čandek et al. 2013, 2015, Kuntner 1996, 1997c, 1997d, Kuntner and Kostanjšek 2000, Kuntner et al. 2008

= *Meta reticulata* (Linnaeus, 1758)

Polenec 1957, 1958, 1960, 1964a, 1969a, 1969b, 1970a, 1970b, 1971a, 1973c, 1978b, 1980a, 1989

= *Meta retuculata typica* 0

Polenec 1957

= *Meta segmentata* (Clerck, 1758)

Nikolić and Polenec 1981, Novak 1981, 2005b, Polenec 1961b, 1975b, 1985

***Pachygnatha clercki*** Sundevall, 1823

Kuntner 1997d, Nikolić and Polenec 1981, Polenec 1957, 1958, 1973c, 1989, 1992

***Pachygnatha degeeri*** Sundevall, 1830

Bole et al. 1980, 1982, Čandek et al. 2013, Gorjan 2014, Gregorič and Kuntner 2009, Kuntner 1999b, Nikolić and Polenec 1981, Polenec 1957, 1958, 1970a, 1973c, 1974a, 1975a, 1975b, 1976b, 1977, 1984, 1986, 1989

= *Aranea degeerii* Scopoli, 1763

Scopoli 1763

***Pachygnatha listeri*** Sundevall, 1830

Bole et al. 1982, Čandek et al. 2013, Kuntner 1997c, 1999b, Nikolić and Polenec 1981, Polenec 1957, 1958, 1960, 1973c, 1975b, 1989

***Pachygnatha terilis*** Thaler, 1991

Kuntner 1997d, 2001, 2002, ULRS 2002

***Tetragnatha extensa*** (Linnaeus, 1758)

Čandek et al. 2013, Kostanjšek 2004a, Kuntner 1996, 1997d, 1999b, Nikolić and Polenec 1981, Polenec 1957, 1958, 1973c, 1989, Tarman 2003

= *Tetragnatha solandri* Scopoli, 1763

Polenec 1954

***Tetragnatha montana*** Simon, 1874

Budja 2008, Kostanjšek 2004a, Kuntner 1996, 1997c, 1997d, 1999b, Polenec 1971c, 1989

***Tetragnatha nigrita*** Lendl, 1886

Čandek et al. 2013, Kuntner 1997d, 2002

***Tetragnatha obtusa*** C. L. Koch, 1837

Kuntner 1997c, 2002, Kuntner et al. 2008, Novak 2005b

***Tetragnatha pinicola*** L. Koch, 1870

Čandek et al. 2013, Kostanjšek 2002b, 2004a, Kuntner 1997c, 1997d, Nikolić and Polenec 1981, Polenec 1957, 1958, 1965a, 1969b, 1971a, 1973c, 1975b, 1989, 1992

**Family THERIDIIDAE**

***Anelosimus vittatus*** (C. L. Koch, 1836)

Agnarsson 2004, Gregorič and Kuntner 2009, Kuntner 2002, Kuntner and Kostanjšek 2000, Le Peru 2011

***Asagena italica*** (Knoflach, 1996)

= *Steatoda italica* Knoflach, 1996

Knoflach 1996

***Asagena phalerata*** (Panzer, 1801)

Čandek et al. 2013, Polenec 1964b, 1970a, 1970b, 1971a, 1989

= *Steatoda phalerata* (Panzer, 1801)

Gregorič and Kuntner 2009, Knoflach 1996, Le Peru 2011, Nikolić and Polenec 1981

***Crustulina guttata*** (Wider, 1834)

Agnarsson 2004, Čandek et al. 2013, Knoflach 1994, Le Peru 2011, Nikolić and Polenec 1981, Polenec 1957, 1958, 1960, 1963e, 1964b, 1965b, 1969a, 1973c, 1978d, 1989

***Crustulina scabripes*** Simon, 1881

Čandek et al. 2013, Gregorič and Kuntner 2009

= *Crustulina scabrosa* 0

Bole et al. 1980

Cryptachaea riparia* (Blackwall, 1834)

1♀ - river bank at the confluence of Cerknica and Idrijca rivers; 46°06,12'N, 13°56,86'E, 240 m a. s. l.; 30.7.2000; leg & det. Kostanjšek R.

1♀ - shaft near house, Dragočajna 4A, Smeldnik; 46°10,41'N, 14°25,08'E, 360 m a. s. l.; 28.7.2003; leg. Štilec K., det. Knapič T., Gregorič M.

2♀ - forest, Krakovski gozd, 2,2 km N from Kostanjevica na Krki; 45°51,82'N, 15°25,28'E, 150 m a. s. l.; 26.7.2009; leg & det. Kostanjšek R.

1♂ - meadow, 700 m NE from Modrejce village; S of Tolmin; 46°10,14'N, 13°45,08'E, 160 m a. s. l.; 22.7.2010; leg & det. Kostanjšek R.

1♂ - Pond Ribnik NW of village Griblje; 45°34,56'N, 15°17,01'E, 150 m a. s. l.; 29.07.2001; leg. & det. Kostanjšek R.

1♀ - Meadow on slope E of Konjsko brdo, Davča, Cerkno; 46°10,44'N, 13°59,49'E,1310 m a. s. l.; 29.07.2000; leg. & det. Kostanjšek R.

***Dipoena braccata*** (C. L. Koch, 1841)

Kostanjšek 2004a, Le Peru 2011

***Dipoena coracina*** (C. L. Koch, 1837)

Le Peru 2011, Nikolić and Polenec 1981, Polenec 1957, 1958

***Dipoena melanogaster*** (C. L. Koch, 1837)

Čandek et al. 2013, Kuntner 1996, 1997d, Kuntner and Baxter 1997, Le Peru 2011, Nikolić and Polenec 1981, Polenec 1957, 1958, 1973c, 1989

***Dipoena torva*** (Thorell, 1875)

Agnarsson 2004, Kuntner 2002, Kuntner and Kostanjšek 2000, Le Peru 2011

***Enoplognatha afrodite*** Hippa & Oksala, 1983

Čandek et al. 2013

***Enoplognatha latimana*** Hippa & Oksala, 1982

Čandek et al. 2013, Kostanjšek 2004a, Kuntner 1997d, 1999b, 2002, Kuntner and Kostanjšek 2000, Le Peru 2011

***Enoplognatha ovata*** (Clerck, 1757)

Čandek et al. 2013, Kostanjšek 2004a, Kuntner 1997c, 1997d, 1999b, Kuntner and Baxter 1997, Kuntner and Kostanjšek 2000, Le Peru 2011, Novak 2005b, Tarman 2003

= *Enoplognatha lineata* (Clerck, 1757)

Budja 2008, Kuntner 1996, Nikolić and Polenec 1981

= *Theridium redimitum* (Linnaeus, 1758)

Polenec 1957, 1958, 1963d, 1967b, 1969b, 1971a, 1973c, 1973e, 1980a, 1985, 1989

***Enoplognatha thoracica*** (Hahn, 1833)

Gorjan 2014, Gregorič and Kuntner 2009, Kuntner 1997d, Le Peru 2011, Nikolić and Polenec 1981, Polenec 1963e, 1974b, 1989, 1992

***Episinus angulatus*** (Blackwall, 1836)

Čandek et al. 2013

***Episinus cavernicola*** (Kulczyński, 1897)

Le Peru 2011

= *Plocamis cavernicola* Kulczyński, 1897

Nikolić and Polenec 1981

***Episinus maculipes*** Cavanna, 1876

Agnarsson 2004, Čandek et al. 2013, Kuntner 1996, 1997a, 2002, Kuntner and Baxter 1997, Kuntner and Kostanjšek 2000, Le Peru 2011

***Episinus truncatus*** Latreille, 1809

Bole et al. 1980, Budja 2008, Čandek et al. 2013, Gorjan 2014, Knoflach and Thaler 2000, Kuntner 1996, 1997c, 1997d, 1999b, Kuntner and Baxter 1997, Kuntner and Kostanjšek 2000, Le Peru 2011, Nikolić and Polenec 1981, Polenec 1957, 1958, 1959, 1961d, 1964a, 1965b, 1978d, 1985, 1989

***Euryopis flavomaculata*** (C. L. Koch, 1836)

Bole et al. 1980, Čandek et al. 2013, Gorjan 2014, Gregorič and Kuntner 2009, Le Peru 2011, Nikolić and Polenec 1981, Polenec 1957, 1958, 1963e, 1973c, 1977, 1981, 1985, 1989

***Euryopis laeta*** (Westring, 1861)

Kostanjšek and Gorjan 2013

***Heterotheridion nigrovariegatum*** (Simon, 1873)

Čandek et al. 2013

= *Theridion nigrovariegatum* Simon, 1873

Kostanjšek 2004b

***Lasaeola prona*** (Menge, 1868)

Kostanjšek and Gorjan 2013

***Lasaeola tristis*** (Hahn, 1833)

= *Dipoena tristis* (Hahn, 1831)

Kuntner 1997d, Le Peru 2011, Nikolić and Polenec 1981, Polenec 1957, 1958, 1973c, 1989, 1992

***Neottiura bimaculata*** (Linnaeus, 1767)

Agnarsson 2004, Arnedo et al. 2004, Čandek et al. 2013, Gorjan 2014, Kuntner 1999b, 2002, Kuntner and Kostanjšek 2000, Le Peru 2011

= *Theridion bimaculatum* (Linnaeus, 1767)

Kuntner 1997d

***Neottiura herbigrada*** (Simon, 1873)

Čandek et al. 2013

***Neottiura suaveolens*** (Simon, 1879)

Čandek et al. 2013, Gorjan 2014, Kuntner 2002, Kuntner and Kostanjšek 2000, Le Peru 2011

***Paidiscura pallens*** (Blackwall, 1834)

Čandek et al. 2013, Knoflach and Thaler 2000, Vanuytven et al. 1994

= *Theridion pallens* Blackwall, 1834

Budja 2008, Kuntner 1997d, Le Peru 2011, Nikolić and Polenec 1981, Polenec 1957, 1958

***Parasteatoda lunata*** (Clerck, 1757)

Čandek et al. 2013

= *Achaearanea lunata* (Clerck, 1757)

Budja 2008, Kuntner 1996, 1997d, 1999b, Kuntner and Baxter 1997, Kuntner and Kostanjšek 2000, Le Peru 2011, Nikolić and Polenec 1981

= *Theridion lunatum* C. L. Koch, 1841

Polenec 1957

= *Theridium lunatum* (Olivier, 1789)

Kratochvíl 1934, Polenec 1958, 1963d, 1964a, 1989

***Parasteatoda simulans*** (Thorell, 1875)

= *Achaearanea simulans* (Thorell, 1875)

Budja 2008, Kuntner 1997d, 1999b, 2002, Le Peru 2011

***Parasteatoda tepidariorum*** (C. L. Koch, 1841)

Čandek et al. 2013

= *Achaearanea tepidariorum* (C. L. Koch, 1841)

Kostanjšek 2004a, Kuntner and Kostanjšek 2000, Le Peru 2011, Novak 2005b

= *Theridion tepidariorum* C. L. Koch, 1841

Polenec 1989

***Pholcomma gibbum*** (Westring, 1851)

Bole et al. 1980, Gorjan 2014, Le Peru 2011, Nikolić and Polenec 1981, Polenec 1976b, 1989

***Phylloneta impressa*** (L. Koch, 1881)

= *Theridion impressum* L. Koch, 1881

Kuntner 1997d, 1999b, Kuntner and Kostanjšek 2000, Le Peru 2011, Nikolić and Polenec 1981

= *Theridium impressum* L. Koch, 1881

Polenec 1971a, 1975b, 1981, 1989

***Phylloneta sisyphia*** (Clerck, 1757)

= *Theridion sisyphium* (Clerck, 1757)

Le Peru 2011, Nikolić and Polenec 1981, Tarman 2003

= *Theridium notatum* Linnaeus

Polenec 1963c, 1964a, 1965a, 1967b, 1969b, 1971a, 1989

***Platnickina tincta*** (Walckenaer, 1802)

Čandek et al. 2013

= *Theridion tinctum* (Walckenaer, 1802)

Kostanjšek 2002b, Kuntner 1996, 1997c, 1997d, 1999a, Kuntner and Baxter 1997, Kuntner and Kostanjšek 2000, Le Peru 2011, Nikolić and Polenec 1981, Polenec 1960, 1989

= *Theridium tinctum* C. L. Koch

Kuntner 1999b, Polenec 1957, 1958, 1960

***Robertus lividus*** (Blackwall, 1836)

Gregorič and Kuntner 2009, Le Peru 2011, Nikolić and Polenec 1981, Polenec 1960, 1961d, 1962a, 1963e, 1964a, 1965b, 1968a, 1969a, 1969c, 1970b, 1974a, 1974b, 1975b, 1977, 1980b, 1984, 1989

***Robertus neglectus*** (O. P.-Cambridge, 1871)

Le Peru 2011, Nikolić and Polenec 1981, Polenec 1957, 1958

***Robertus scoticus*** Jackson, 1914

Čandek et al. 2013

***Robertus truncorum*** (L. Koch, 1872)

Bole et al. 1980, Le Peru 2011, Nikolić and Polenec 1981, Polenec 1979a, 1981, 1989

***Rugathodes bellicosus*** (Simon, 1873)

= *Theridion bellicosum* (Simon, 1873)

Polenec 1968a, 1992

= *Theridion instabile bellicosum* (Simon, 1873)

Nikolić and Polenec 1981

= *Theridium bellicosum* Simon, 1873

Polenec 1970a, 1971b, 1973b, 1989

***Sardinidion blackwalli*** (O. P.-Cambridge, 1871)

Čandek et al. 2013

= *Theridion blackwalli* Cambridge, 1870

Kuntner 1999a, 1999b, Kuntner and Kostanjšek 2000, Le Peru 2011, Nikolić and Polenec 1981, Polenec 1989, 1992

= *Theridium blackwalli* Cambridge, 1870

Polenec 1958, 1988

***Simitidion simile*** (C. L. Koch, 1836)

Čandek et al. 2013, Kostanjšek 2003

= *Theridion simile* C. L. Koch, 1836

Le Peru 2011, Nikolić and Polenec 1981, Polenec 1992

= *Theridium simile* C. L. Koch, 1836

Polenec 1957, 1958, 1973c, 1989

***Steatoda bipunctata*** (Linnaeus, 1758)

Agnarsson 2004, Čandek et al. 2013, Kostanjšek 2004a, Kuntner 1996, 1997d, 1999b, Kuntner and Kostanjšek 2000, Le Peru 2011, Nikolić and Polenec 1981, Polenec 1962b, 1966b, 1968a, 1989, Tarman 2003

***Steatoda grossa*** (C. L. Koch, 1838)

Le Peru 2011, Nentwig and Kobelt 2010, Nikolić and Polenec 1981

***Steatoda paykulliana*** (Walckenaer, 1805)

Kuntner 1997d, Le Peru 2011, Polenec 1966a

= *Lithyphantes paykullianus* (Walckenaer, 1805)

Nikolić and Polenec 1981, Polenec 1969a

***Steatoda triangulosa*** (Walckenaer, 1802)

Čandek et al. 2013, Kuntner 1999b, 2002, Kuntner and Kostanjšek 2000, Le Peru 2011, Nentwig and Kobelt 2010

***Theridion betteni*** Wiehle, 1960

Kostanjšek 2002a, 2004a

Theridion boesenbergi* Strand, 1904

20♂, 166♀ - primary forest, Rajhenavski Rog sanctuary, Kočevje region; 45°41'N, 15°01'E, 870-920 m a. s. l.; 25-28.6.1999; leg. Floren A., det. Agnarsson I.

Theridion familiare* O. P.-Cambridge, 1871

1♀ - primary forest, Rajhenavski Rog sanctuary, Kočevje region; 45°41'N, 15°01'E, 870-920 m a. s. l.; 25-28.6.1999; leg. Floren A., det. Agnarsson I.

***Theridion melanurum*** Hahn, 1831

Kostanjšek 2003, Le Peru 2011, Nikolić and Polenec 1981

= *Theridium denticulatum* Walckenaer

Polenec 1958

***Theridion mystaceum*** L. Koch, 1870

Budja 2008, Kuntner 2002, Le Peru 2011

***Theridion petraeum*** L. Koch, 1872

Le Peru 2011, Nikolić and Polenec 1981

= *Theridium petraeum* L. Koch, 1872

Kratochvíl 1934

***Theridion pictum*** (Walckneaer, 1802)

Vanuytven et al. 1994

***Theridion pinastri*** L. Koch, 1872

Budja 2008, Čandek et al. 2013, Kostanjšek 2002b, Kuntner 1997c, 2002, Kuntner and Kostanjšek 2000, Le Peru 2011

***Theridion varians*** Hahn, 1833

Agnarsson 2004, Budja 2008, Čandek et al. 2013, Kuntner 1997c, 1997d, Kuntner and Kostanjšek 2000, Le Peru 2011, Nikolić and Polenec 1981, Polenec 1989

= *Theridium varians* Hahn, 1833

Kuntner 1999b, Polenec 1957, 1958, 1963d, 1974a

***Theridula gonygaster*** (Simon, 1873)

Kostanjšek 2010, 2011, 2012

**Family THERIDIOSOMATIDAE**

***Theridiosoma gemmosum*** (L. Koch, 1877)

Kuntner 1999b, 2001, 2002, Le Peru 2011, ULRS 2002

**Family THOMISIDAE**

***Coriarachne depressa*** (C. L. Koch, 1837)

Nikolić and Polenec 1981, Polenec 1957, 1958, 1962a, 1965b, 1968b, 1973c, 1989

***Cozyptila blackwalli*** (Simon, 1875)

= *Oxyptila blackwalli* Simon, 1875

Bole et al. 1980, Nikolić and Polenec 1981, Polenec 1976b, 1978d, 1989, Šilhavy 1944

= *Ozyptila blackwalli* Simon, 1875

Gorjan 2014

***Diaea dorsata*** (Fabricius, 1777)

Budja 2008, Kuntner 1996, 1997c, 1997d, 1999b, Kuntner and Kostanjšek 2000, Nikolić and Polenec 1981, Novak 1981, 2005b, Polenec 1957, 1958, 1959, 1960, 1964a, 1965a, 1969b, 1970a, 1970b, 1973c, 1975b, 1986, 1989, Tarman 2003

***Diaea livens*** Simon, 1876

Čandek et al. 2013

***Ebrechtella tricuspidata*** (Fabricius, 1775)

Čandek et al. 2013

= *Misumenops tricuspidatus* (Fabricius, 1775)

Budja 2008, Kostanjšek 2004a, Kuntner 1996, 1997a, 1997c, 1997d, 2002

***Heriaeus hirtus*** (Latreille, 1819)

Čandek et al. 2013, Kostanjšek 2010

***Misumena vatia*** (Clerck, 1757)

Budja 2008, Čandek et al. 2013, Kostanjšek 2003, 2004a, Kuntner 1996, 1997c, 1997d, 1999b, Kuntner and Kostanjšek 2000, Nikolić and Polenec 1981, Polenec 1984, 1989, Tarman 2003, Vogrin 2005

= *Misumena calycina* Linnaeus

Polenec 1957, 1958, 1963a, 1964a, 1965a, 1969b, 1971a, 1971c, 1973c, 1973e, 1980a, 1985

***Ozyptila atomaria*** (Panzer, 1801)

Gorjan 2014, Gregorič and Kuntner 2009

= *Oxyptila atomaria* (Panzer, 1801)

Bole et al. 1980, Nikolić and Polenec 1981, Polenec 1961d, 1962a, 1965a, 1968b, 1969a, 1975a, 1975b, 1977, 1981, 1985, 1986, 1989

= *Oxyptila horticola* (C. L. Koch, 1837)

Polenec 1957, 1958, 1963e, 1973c, 1989, 1992

***Ozyptila brevipes*** (Hahn, 1826)

= *Oxyptila brevipes* (Hahn, 1826)

Polenec 1957, 1958, 1992

***Ozyptila claveata*** (Walckenaer, 1837)

Čandek et al. 2013, Gorjan 2014

= *Oxyptila nigrita* (Thorell, 1875)

Bole et al. 1980, Polenec 1992

Ozyptila praticola* (C. L. Koch, 1837)

1♀ - forest, 3 km S of Rače in NE Slovenia; 46°25,73'N, 15°40,83'E, 250 m a. s. l.; 18.7.2013; leg. & det. Kostanjšek R.

5♂ - forest, 1 km N of Marjeta na Dravskem polju, E of Rače in NE Slovenia; N 46°27,00'N, 15°43,32'E, 250 m a. s. l.; 18.7. - 24.7.2013; leg. Kostanjšek & det. Kostanjšek R.

1♀ - forest, Kozlarjeva gošča, Ljubljansko barje marsh, S of Ljubljana; 45°59,64'N, 14°30,28'E, 290 m a. s. l.; 28.9.2002; leg. Vrezec A, & det. Fišer C., Kostanjšek R.

***Ozyptila pullata*** (Thorell, 1875)

= *Oxyptila kotulai* Kulczyński, 1898

Polenec 1992

***Ozyptila rauda*** Simon, 1875

= *Oxyptila rauda* Simon, 1875

Nikolić and Polenec 1981, Polenec 1973e, 1992

***Ozyptila scabricula*** (Westring, 1851)

= *Oxyptila scabricula* (Westring, 1851)

Nikolić and Polenec 1981, Polenec 1962a, 1968b, 1969a, 1975a

***Ozyptila simplex*** (O. P.-Cambridge, 1862)

Gorjan 2014

= *Oxyptila simplex* (Cambridge, 1862)

Bole et al. 1980, Polenec 1986, 1989, 1992

***Ozyptila trux*** (Blackwall, 1846)

Kuntner and Kostanjšek 2000

= *Oxyptila trux* (Blackwall, 1846)

Bole et al. 1980, Nikolić and Polenec 1981, Polenec 1978c, 1982a, 1989

***Pistius truncatus*** (Pallas, 1772)

Gorjan 2014, Kuntner 1996, 1997a, 1999b, 2002, Kuntner and Kostanjšek 2000

***Runcinia grammica*** (C. L. Koch, 1837)

Kostanjšek 2005, 2010

***Synema globosum*** (Fabricius, 1775)

Čandek et al. 2013, Kostanjšek 2004a, Kuntner 1996, 1997c, 1997d, 1999b, Kuntner and Kostanjšek 2000, Nikolić and Polenec 1981, Polenec 1957, 1958, 1973c, 1989

***Thomisus onustus*** Walckenaer, 1805

Čandek et al. 2013, Kuntner 1996, 1997d, Kuntner and Kostanjšek 2000, Nikolić and Polenec 1981

***Tmarus piger*** (Walckenaer, 1802)

Čandek et al. 2013, Gorjan 2014, Kostanjšek 2003, Kuntner 1997d, 1999b, Kuntner and Kostanjšek 2000, Nikolić and Polenec 1981, Polenec 1957, 1958, 1960, 1969a, 1973c, 1978d, 1989

***Tmarus stellio*** Simon, 1875

Kuntner 1997d, 1999b, 2002

***Xysticus acerbus*** Thorell, 1872

Čandek et al. 2013, Gorjan 2014, Gregorič and Kuntner 2009, Kostanjšek 2003

***Xysticus audax*** (Schrank, 1803)

Bole et al. 1980, Čandek et al. 2013, Gorjan 2014, Kuntner 1997c, Nikolić and Polenec 1981, Polenec 1986, 1989

= *Xysticus pini* Hahn

Polenec 1957, 1958, 1959, 1962a, 1965b, 1970a, 1971a, 1973c

= *Xysticus pini audax* Hahn

Polenec 1973e

***Xysticus bifasciatus*** C. L. Koch, 1837

Bole et al. 1980, Čandek et al. 2013, Gregorič and Kuntner 2009, Kostanjšek 2004a, Nikolić and Polenec 1981, Polenec 1970a, 1970b, 1975b, 1976b, 1978c, 1980b, 1981, 1982a, 1984, 1986, 1989, Šilhavy 1944, Tarman 2003

***Xysticus bufo*** (Dufour, 1820)

= *Xysticus albimanus* Simon, 1932

Polenec 1992

***Xysticus cristatus*** (Clerck, 1757)

Budja 2008, Čandek et al. 2013, Gregorič and Kuntner 2009, Kostanjšek 2003, 2004a, Kuntner 1997c, 1997d, Nikolić and Polenec 1981, Polenec 1963e, 1978b, 1980b, 1982a, 1984, 1985, 1989

= *Xysticus viaticus* Linnaeus

Polenec 1958, 1962a, 1971a

***Xysticus desidosus*** Simon, 1875

Čandek et al. 2013

***Xysticus erraticus*** (Blackwall, 1834)

Bole et al. 1980, Čandek et al. 2013, Gorjan 2014, Gregorič and Kuntner 2009, Kuntner 1997d, Nikolić and Polenec 1981, Polenec 1962a, 1968b, 1969a, 1973d, 1975a, 1980b, 1981, 1984, 1989

***Xysticus ferrugineus*** Menge, 1876

Bole et al. 1980, Polenec 1992

***Xysticus gallicus*** Simon, 1875

Bole et al. 1980, Gorjan 2014, Nikolić and Polenec 1981, Polenec 1977, 1978c, 1981, 1983, 1986, 1989

***Xysticus kempeleni*** Thorell, 1872

Čandek et al. 2013

***Xysticus kochi*** Thorell, 1872

Bole et al. 1980, Čandek et al. 2013, Gorjan 2014, Gregorič and Kuntner 2009, Kostanjšek 2003, 2004a, Kuntner 1999b, Polenec 1980b, 1981, 1986, 1989

***Xysticus lanio*** C. L. Koch, 1835

Bole et al. 1980, Caporiacco 1949, Čandek et al. 2013, Gorjan 2014, Kostanjšek 2003, Kuntner 1997d, Nikolić and Polenec 1981, Polenec 1957, 1958, 1963e, 1969b, 1970a, 1985, 1989

***Xysticus lineatus*** (Westring, 1851)

Bole et al. 1980, Čandek et al. 2013, Nikolić and Polenec 1981, Polenec 1957, 1958, 1980b, 1981

***Xysticus luctuosus*** (Blackwall, 1836)

Gorjan 2014, Polenec 1980b, 1989, 1992

***Xysticus marmoratus*** Thorell, 1875

= *Xysticus embriki* Kolosváry, 1935

Nikolić and Polenec 1981, Polenec 1992

***Xysticus ninnii*** Thorell, 1872

Kostanjšek 2010, 2011

***Xysticus robustus*** (Hahn, 1832)

Bole et al. 1980, Gregorič and Kuntner 2009, Kuntner 1997d, Polenec 1963e, 1965b, 1966a, 1978d, 1983, 1989

= *Proxysticus robustus* (Hahn, 1831)

Nikolić and Polenec 1981

***Xysticus sabulosus*** (Hahn, 1832)

Kostanjšek 2004a

= *Xysticus cambridgei* (Blackwall, 1859)

Polenec 1986, 1992

***Xysticus striatipes*** L. Koch, 1870

Gorjan 2014, Gregorič and Kuntner 2009, Polenec 1992

***Xysticus tenebrosus*** Silhavy, 1944

Čandek et al. 2013

***Xysticus ulmi*** (Hahn, 1831)

Kostanjšek 2004a, Kuntner 1996, 1999b, 2002

**Family TITANOECIDAE**

***Nurscia albomaculata*** (Lucas, 1846)

Kostanjšek 2005, 2010

***Titanoeca quadriguttata*** (Hahn, 1833)

Polenec 1989, 1992

= *Titanoeca obscura* 0

Bole et al. 1982

***Titanoeca tristis*** L. Koch, 1872

Čandek et al. 2013

***Titanoeca veteranica*** Herman, 1879

Gregorič and Kuntner 2009, Polenec 1992

**Family TRACHELIDAE**

***Paratrachelas maculatus*** (Thorell, 1875)

= *Trachelas maculatus* Thorell, 1875

Kuntner 1997a, 2001, 2002, ULRS 2002

**Family ULOBORIDAE**

***Hyptiotes paradoxus*** (C. L. Koch, 1834)

Budja 2008, Čandek et al. 2013, Kostanjšek 2007, Kuntner 1996, 1997c, 1999b, Kuntner and Baxter 1997, Kuntner and Kostanjšek 2000, Le Peru 2011, Polenec 1958, 1961c, 1964b, 1967b, 1989

= *Uptiotes paradoxus* (C. L. Koch, 1877)

Nikolić and Polenec 1981

***Uloborus plumipes*** Lucas, 1846

Čandek et al. 2013, Kuntner 1994, 2002, Tarman 2003

***Uloborus walckenaerius*** Latreille, 1806

Čandek et al. 2013, Kuntner 2002, Le Peru 2011, Polenec 1992, Tarman 2003

**Family ZODARIIDAE**

***Zodarion germanicum*** (C. L. Koch, 1837)

Nikolić and Polenec 1981, Polenec 1968a, 1970a, 1989, 1992

= *Zodarium germanicum* C. L. Koch, 1837

Polenec 1961d, 1965a

***Zodarion hamatum*** Wiehle, 1964

Bole et al. 1982, Bosmans 1997, Gorjan 2014, Gregorič and Kuntner 2009, Nikolić and Polenec 1981, Polenec 1969a, 1978d, Tarman 2003, Wiehle 1964, Wunderlich 1980

***Zodarion italicum*** (Canestrini, 1868)

Kuntner 2001, Polenec 1992, ULRS 2002, Wunderlich 1980

***Zodarion pusio*** Simon, 1914

Kostanjšek and Gorjan 2013

***Zodarion scutatum*** Wunderlich, 1980

Bosmans 2009, Kuntner 2001, Mršić 1997, Polenec 1992, ULRS 2002, Wunderlich 1980

**Family ZOROPSIDAE**

***Zoropsis spinimana*** (Dufour, 1820)

Kostanjšek and Kuntner 2002, Kuntner 2001, Kuntner and Kostanjšek 2000, Nikolić and Polenec 1981

### Redeterminations and omissions from the list

Family Amaurobiidae

*Amaurobius pallidus* L. Koch, 1868

The single specimen recorded (Kostanjšek 2005) was misidentified and the species should be omitted from the list.

Family Araneidae

*Argiope lobata* (Pallas, 1772)

The author (Polenec 1952) affiliates the species to Primorje, which in this case refers to coastal region of the former Yugoslavia. However, the species has never been recorded in Slovenia.

*Araniella displicata* (Hentz, 1847)

The single specimen (Kostanjšek 2005) was re-determined as *Araniella cucurbitina* (Clerck, 1757) by the author of the original paper.

Family Cybaeidae

*Cybaeus angustiarum* L. Koch, 1868

As the species is restricted to eastern parts of Central Europe, East and South-East Europe (Maurer 1992), the single specimen listed in Novak (2005b) most likely belongs to *Cybaeus tetricus* (C. L. Koch, 1839).

Family Dictynidae

*Lathys nielseni* (Schenkel, 1932)

The specimen listed in Čandek et al. (2013) has been re-determined as *Lathys humilis* (Blackwall, 1855) (Čandek et al. 2015).

Family Dysderidae

*Dysdera erythrina* (Walckenaer, 1802)

The specimen listed in Čandek et al. (2013) has been re-determined as *Dysdera adriatica* (Kulczyński, 1897) (Čandek et al. 2015).

Family Gnaphosidae

*Callilepis nocturna* (Linnaeus, 1758)

The single specimen (Kostanjšek 2005) was re-determined as *Callilepis schuszteri* (Herman, 1879) by the author of the original paper.

Family Liocranidae

*Scotina gracilipes* (Blackwall, 1859)

The single record of the species in Slovenia (Polenec 1989) appears as “*Scotina palliardi* Reimoser, 1937 syn. *Scotina gracillipes* (Blackwall, 1859)”. Since these names are not synonyms (World Spider Catalog 2014) and the presence of *Scotina palliardi* in Slovenia is confirmed by other records (Bole 1980; Polenec 1981), we omitted *Scotina gracilipes* from the list.

Family Lycosidae

*Alopecosa barbipes* (Sundevall, 1833)

The species has an Atlantic distribution (Cordes and von Helversn 1990), and all Slovenian records belong to *Alopecosa accentuata* (Latreille, 1817).

Family Mimetidae

*Ero cambridgei* Kulczyński, 1911

The single specimen (Kostanjšek 2005) was re-determined as *Ero tuberculata* (De Geer, 1778) by the author of the original paper.

Family Uloboridae

*Uloborus glomosus* (Walckenaer, 1841)

The species originally determined as *Uloborus glomosus* (Kuntner 1994) was later re-determined as *Uloborus plumipes*, Lucas, 1846 by the author (Kuntner and Šereg 2002). However, the re-determination was overlooked in a Slovenian fauna overview (Tarman 2003), where *Uloborus glomosus* was falsely listed as introduced species.

Family Salticidae

*Pellenes tripunctatus* (Walckenaer, 1802)

The specimen listed in Čandek et al. (2013) has been re-determined as *Pellenes seriatus* (Thorell, 1875) (Čandek et al. 2015).

*Sitticus zimmermanni* (Simon, 1877)

The species encompass two attitudinally segregated species (Kronestedt and Logunov 2003) and since all Slovenian specimens were collected in alpine environments, the records belong to *Sitticus atricapillus* (Simon, 1882).

### Unconfirmed records

**Unconfirmed records:** The following records were listed in the checklist of Yugoslav spiders (Nikolić and Polenec 1981) as expected in Slovenia, however their presence has not been confirmed.

*Agyneta arietans* (Cambridge, 1872)
*Allomengea scopigera* (Grube, 1859)
*Altella lucida* (Simon, 1874)
*Anacotyle austera* (Simon, 1884)
*Araneus carbonarus* (L. Koch, 1869)
*Araneus ocellatus* Clerck, 1757
*Bolyphantes crucifer* (Menge, 1866)
*Bolyphantes index* (Thorell, 1856)
*Bolyphantes nigropictus* Simon, 1884
*Cheiracanthium oncognathum* Thorell, 1871
*Cheiracanthium seidlitzii* L. Koch, 1864
*Clubiona corticalis nigra* Simon, 1878
*Coelotes pabulator* Simon, 1875
*Coelotes simoni* Strand, 1907
*Dictyna sedilloti* Simon, 1875
*Dipoena convexa* (Blackwall, 1870)
*Dismodicus bifrons* (Blackwall, 1834)
*Drassodes canaglensis* Di Caporiacco, 1926
*Drassodes dificilis* (Simon, 1878)
*Drassodes fugax* (Simon, 1878)
*Drassodes luteomicans* (Simon, 1878)
*Drassodes lutescens* (C.L. Koch, 1839)
*Drassodes villosus* (Thorell, 1856)
*Drassodes vinosus* (Simon, 1878)
*Dysdera subsquarrosa* Simon, 1914
*Enoplognatha testacea* Simon, 1884
*Erigone atra* Blackwall, 1833
*Erigone remota* L. Koch, 1869
*Euophrys acriceps* (Simon, 1871)
*Euophrys rufibarbis* (Simon, 1868)
*Euophrys sulphurea* (L. Koch, 1867)
*Euophrys testaceozonata* (Capoiracco, 1922)
*Euryopis quinqueguttata* Thorell, 1875
*Gnaphosa petrobia* L. Koch, 1872
*Gongylidiellum latebricola* (Cambridge, 1871)
*Gongylidiellum murcidum* Simon, 1884
*Hahnia muscicola* Simon, 1875
*Haplodrassus umbratilis* (L. Koch, 1866)
*Hylyphantes graminicola* (Sundevall, 1830)
*Kaestneria approximata* (Cambridge, 1871)
*Lephtyphantes culicinus* Simon, 1884
*Lephtyphantes frigidus* Simon, 1884
*Lephtyphantes monachus* Simon, 1884
*Lephtyphantes pulcher* (Kulczyński, 1882)
*Microclubiona diversa* (Cambridge, 1862)
*Neottiura bimaculata pellucida* (Simon, 1873)
*Nothocyba laudata* (Cambridge, 1881)
*Olios argelasius* (Walckenaer, 1805)
*Pardosa paludicola* (Claerck, 1757)
*Pardosa proxima poetica* Simon, 1876
*Pardosa strigilata* Simon, 1876
*Pardosa torrentum* Simon, 1876
*Phlodromus emarginatus* (Schrank, 1803)
*Phlodromus glaucinus* Simon, 1870
*Philodromus vagulus* Simon, 1875
*Poeciloneta variegata* (Blackwall, 1841)
*Scotinotylus antennatus* (Cambridge, 1875)
*Sitticus rupicila* (C. L. Koch, 1837)
*Tegenaria picta* Simon, 1870
*Textris coarctata* (Dufour, 1831)
*Theridion aulicum* C. L. Koch, 1838
*Theridion instabile* Cambridge, 1871
*Theridion musivum* Simon, 1873
*Theridion varians rusticum* (Simon, 1873)
*Trachelas minor* Cambridge, 1872
*Trichoncus sordidus* Simon, 1884
*Xerolycosa albofasciata* (Brulle, 1832)
*Xysticus gortanii* Di Caporiacco, 1922
*Xysticus nubilus* Simon, 1875
*Xysticus parallelus* Simon, 1873
*Zelotes barbatus* (L. Koch, 1866)
*Zelotes civicus* (Simon, 1878)
*Zelotes talpinus* (L. Koch, 1866)

### Nomina dubia

Family Hahniidae

*Cryphoeca cavicola* Caporiacco, 1938 (Brignoli 1976; Nikolić and Polenec 1981; Polenec 1978d)

Family Philodromidae

*Philodromus corticinus* (C. L. Koch, 1837) (Kostanjšek 2004b)

Family Linyphiidae

*Porrhomma fonsfrigidum* Drensky, 1929 (Kratochvíl 1936; Nikolić and Polenec 1981)

*Porrhomma calypso* (Bertkau, 1883) (Nikolić and Polenec 1981)

Family Gnaphosidae

*Zelotes ater* (Hentz, 1832) listed in (Nikolić and Polenec 1981)

### Nomina nuda

The following genus-species combinations used by authors in parentheses have not been used in any taxonomic literature.

*Oxyptila blanckardii* (Bole 1980)

*Gnaphosa bifasciata* (Bole 1980)

*Aranea clerkii* (Scopoli 1763)

*Aranea derhamii* (Scopoli 1763)

*Aranea forskaelii* (Scopoli 1763)

*Aranea goedarti* (Scopoli 1763)

*Aranea hasselquistii* (Scopoli 1763)

*Aranea hombergii* (Scopoli 1763)

*Aranea jonstoni* (Scopoli 1763)

*Aranea kalmii* (Scopoli 1763)

*Aranea kleinii* (Scopoli 1763)

*Aranea knorrii* (Scopoli 1763)

*Aranea linnaei* (Scopoli 1763)

*Aranea listeria* (Scopoli 1763)

*Aranea lyonetti* (Scopoli 1763)

*Aranea malpighii* (Scopoli 1763)

*Aranea mouffeti* (Scopoli 1763)

*Aranea osbekii* (Scopoli 1763)

*Aranea petiverii* (Scopoli 1763)

*Aranea podae* (Scopoli 1763)

*Aranea roberti* (Scopoli 1763)

*Aranea rolandri* (Scopoli 1763)

*Aranea schaefferi* (Scopoli 1763)

*Aranea solandri* (Scopoli 1763)

*Aranea uddmanni* (Scopoli 1763)

*Aranea wilkii* (Scopoli 1763)

*Aranea sebae* (Scopoli 1772)

*Lepthyphantes berolinensis* (Polenec 1992)

## Conclusions

We provided a critical review of all literature relevant for the Slovenian spider fauna, and provided the first comprehensive national species list. This tool will fill the void in cataloguing regional European spider faunas and allow country wide comparisons (Kuntner and Šereg 2002). Additionally, it will facilitate further araneological research in Slovenia, perhaps solidifying the already existing trend for a shift away from strictly descriptive biology towards large scale revisionary, phylogenetic, ecological, biogeographical and experimental science. Such a shift would reinforce this species list’s utility and value as baseline biological data.
